# Mapping the Flammability Space of Sustainable Refrigerant Mixtures through an Artificial Neural Network Based on Molecular Descriptors

**DOI:** 10.1021/acssuschemeng.4c01961

**Published:** 2024-07-23

**Authors:** Carlos G. Albà, Ismail I. I. Alkhatib, Lourdes F. Vega, Fèlix Llovell

**Affiliations:** † Department of Chemical Engineering, ETSEQ, Universitat Rovira i Virgili (URV), Campus Sescelades, Av. Països Catalans 26, 43007 Tarragona, Spain; ‡ Research and Innovation Center on CO_2_ and Hydrogen (RICH Center) and Department of Chemical and Petroleum Engineering, Khalifa University, PO Box 127788 Abu Dhabi, United Arab Emirates

**Keywords:** artificial neural networks, flammability, low-GWP refrigerants, applicability domain, normalized flammability index, industry cooling demands

## Abstract

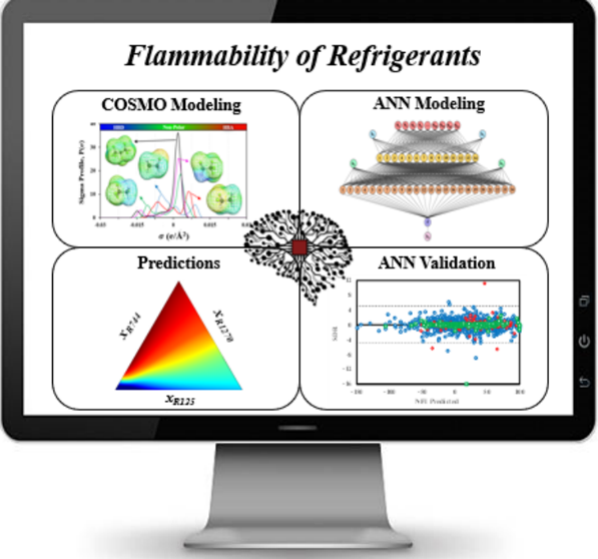

As the EU’s mandates to phase out high-GWP refrigerants come into effect, the refrigeration industry is facing a new, unexpected reality: the introduction of more flammable yet environmentally compliant alternatives. This paradigm shift amplifies the need for a rapid, reliable screening methodology to assess the propensity for flammability of emerging fourth generation blends, offering a pragmatic alternative to laborious and time-intensive traditional experimental assessments. In this study, an artificial neural network (ANN) is meticulously constructed, evaluated, and validated to address this emerging challenge by predicting the normalized flammability index (NFI) for an extensive array of pure, binary, and ternary mixtures, reflecting a substantial diversity of compounds like CO_2_, hydrofluorocarbons (HFCs), hydrofluoroolefins (HFOs), six saturated hydrocarbons (sHCs), hydroolefins (HOs), and others. The optimal configuration ([61 (I) × 14 (HL1) × 24 (HL2) × 1 (O)]) demonstrated a profound fit to the data, with metrics like *R*^2^ of 0.999, root-mean-square error (RMSE) of 0.1735, average absolute relative deviation (AARD)% of 0.8091, and SD_av_ of ±0.0434. Exhaustive assessments were conducted to ensure the most efficient architecture without compromising the accuracy. Additionally, the analysis of the standardized residuals (SDR) and applicability domain (AD) exhibited fine control and consistency over the data points. External validation using quaternary mixtures further attested to the model’s adaptability and predictive capability. The exploration into the relative contribution of descriptors led to the identification of 23 significant sigma descriptors derived from conductor-like screening model (COSMO), responsible for 90.98% of the total contribution, revealing potential avenues for model simplification without a substantial loss in predictive power. Moreover, the model successfully predicted the behavior of prospective industry-relevant mixtures, reinforcing its reliability and opening the door to experimentation with untested blends. The results collectively manifest the developed ANN’s efficiency, robustness, and adaptability in modeling flammability, catering to the demands of industry standards, environmental concerns, and safety requirements.

## Abstract 2

This study develops an ANN to compute flammability KPIs, aiding sustainable refrigeration by enabling rapid assessment of eco-friendly alternatives.

## Introduction

With the growing concerns surrounding climate change,^1−3^ the regulation and management of refrigerants with high global warming potential (GWP)—have become a focal point of attention in recent years.^4,5^ In this regard, the European Union has led the charge in this direction, aiming to reduce hydrofluorocarbon (HFC) emissions by two-thirds by 2030.^6^

Subsequent amendments have further promoted the use of alternative refrigerants with lower GWP such as hydrofluoroolefins (HFOs), hydrochlorofluoroolefins (HCFOs), saturated hydrocarbons (sHC), hydroolefins (HO), and others, even including the previously phased-out CO_2_.^7−10^ This strategy toward low-GWP refrigerants has emerged not merely as a policy matter, but as an environmental imperative, transforming the pursuit of sustainable solutions from an optional requirement into a mandate.^11^ However, this seemingly positive movement toward environmental compliance has unfolded an unexpected challenge;^12,13^ as the newly formulated refrigerants, whether pure fluids such as 2,3,3,3-tetrafluoropropene (R1234yf), propane (R290), isobutane (R600a), propylene (R1270), or difluoromethane (R32), or blends of at least two refrigerants such as R448A, R449A, while less detrimental to climate change, often exhibit highly flammable characteristics compared to their predecessors. Hydrocarbons are re-emerging^14^ as promising solutions following setbacks in past efforts, and a reevaluation^15,16^ of their potential has brought them back into focus. Known for their very low GWP and zero ozone depletion potential (ODP), light hydrocarbons are both environmentally benign and efficient conductors of heat.^17^ Nevertheless, their use is not without challenges; their flammability has always led to concerns and rigorous safety standards, being typically classified in the high flammability class.^18^ In this sense, the reintroduction of aliphatic hydrocarbon gases in new mixtures of refrigerants aligns with global sustainability goals but faces intricate challenges linked to intrinsic flammability concerns. Overall, this paradigm shift is introducing significant safety challenges^19,20^ into the refrigeration industry of today, potentially elevating the cost of associated equipment and demanding a new level of awareness and preparedness.

Flammability is an essential and complex characteristic of refrigerants, encompassing an array of properties and subject to various standards. According to the ANSI/ASHRAE Standard 34^21^ and ISO Standard 817,^22^ flammability is classified based on a combination of factors, including heat of combustion, lower flammability limit (LFL), and laminar burning velocity. Within these standards, refrigerants are assigned to one of three classes, from class 1, signifying no flame propagation under conditions of 60 °C and 101.3 kPa, to class 3, signifying higher flammability with criteria such as a heat of combustion of >19 MJ/kg or a LFL of less than 0.10 kg/m^3^. A subclass “2L” adds further nuance to class 2, imposing additional restrictions on burning velocity.^23^ However, these distinctions do not always lead to a clear demarcation between flammable and nonflammable substances.^24^ In fact, flammability exists on a continuum,^25^ where substances like propane display notable flammability, others like carbon dioxide are entirely nonflammable, and many substances fall along a spectrum of varying flammability levels in between. Certainly, the multifaceted nature of flammability does not end with classification; predicting flammability is further complicated by various factors,^26,27^ including flame propagation, thermal heat dissipation, and buoyancy, all of which require a deeper understanding. The work by researchers such as Egolfopoulos^28,29^ and Linteris and Babushok^30−32^ highlights the current state of understanding and the areas where more research is needed. This intricate interplay between different properties and influencing factors emphasizes the challenging yet vital nature of grasping flammability in the context of refrigerant science, a pursuit that continuously evolves with the ongoing advancements in the field. The quest for an accurate representation of flammability has led to the adoption of various methodologies and metrics,^23^ among which the normalized flammability index (NFI) has emerged as a significant tool.

In the context of this work, the NFI was used to estimate flammability, and this choice is justified by several key attributes of the index. Unlike some conventional metrics, the NFI takes into account a comprehensive range of factors, incorporating the heat of combustion, lower flammability limit, and laminar burning velocity,^33^ while also allowing for normalization based on a reference substance. This enables a better understanding of flammability, offering insights that reflect the real-world complexity of the phenomenon, depending both on the substance’s properties and the conditions of evaluation, including temperature, pressure, and mixture composition. By integrating these factors into a single index, the NFI succeeds in providing a more cohesive and holistic assessment of flammability, complementing other well-established methods, such as the flame spread index (FSI), flash point index, heat release rate (HRR), material calorific value (MCV), fire propagation index (FPI), and limiting oxygen index (LOI). Historically, the development and utilization of the NFI trace back to 2019, as Linteris et al.^24^ sought more accurate and comprehensive ways to understand and manage the risks associated with flammable substances. In the present-day context, the use of the NFI has expanded far beyond its original purpose, covering various fields within refrigeration, including the design of binary,^34^ ternary,^35,36^ and quartenary^37^ fourth generation drop-ins along with comprehensive testing of different types of circuit configurations.^38^ Further assessment of binary mixtures involving hydrocarbons and A2 or A2L components is presented in the work of Calleja-Anta et al.^39^

Given such an evolving landscape, the need for an efficient, rapid, and reliable screening tool to evaluate the flammability of contemporary drop-in alternatives to third generation HFCs has come to the forefront. This stands in contrast to traditional experimental procedures, which are time-intensive, laborious, and often costly. Though a prior contribution^37^ explored the flammability concerns of these avant-garde refrigerant systems by developing an equation to evaluate the NFI, only a limited view was provided.

Our study embarks on a mission to bridge this gap, presenting a Machine Learning approach based on an artificial neural network (ANN) to accurately predict the ASHRAE designations and safety classifications across a wide range of fourth generation refrigerant blends and novel configurations. ML techniques have become popular for their versatile capability to forecast a multitude of properties, including solubility, thermal and electrical conductivity, surface tension, vapor pressure, pH, density, viscosity, and heat capacity, among others. Notably, recent studies had employed a range of inputs including critical coordinates, acentric factor, vapor pressure, molar mass, number of fluorine atoms, and Lennard-Jones interaction parameters to accurately describe the solubility^40,41^ and liquid density^42^ of F-gases. Building on this potential, Table 1 offers a comprehensive overview of machine learning models in the literature using σ-profiles as inputs, encompassing various families of compounds and thus showcasing the versatility of such predictive approaches. However, our 2022 publication^43^ stands as the sole documented instance in the literature to pioneer the use of machine learning, specifically through ANN, for evaluating F-based refrigerants through conductor-like screening model (COSMO) descriptors, underscoring its unique importance in this domain. This extensive applicability demonstrates ML’s robust potential, opening new horizons for analyzing flammability characteristics.

**Table 1:** Machine Learning Models Developed in the Literature Using Molecular Descriptors as Inputs are Sorted by Year of Publication^a^

refs	compounds family	property assessed	ML method
(48)	IL	density, molar liquid volume	MLR
(49)	solvents	polarizability	RBNN
(50)	IL	density	NN
(51)	IL	toxicological effect	MLP
(52)	organic solvents + IL	solvatochromic parameter	RBNN
(53)	IL	activity, enantio-selectivity	ANN + MLR
(54)	IL	viscosity	MLR + SVM
(55)	IL	heat capacity	MLR + ELM
(56)	IL	H_2_S solubility	QSPR + ELM
(57)	IL	ecotoxicity	MLR + MLP
(58)	IL	refractive index	ELM + MLR
(59)	IL	Henry’s law constant	MLR + SVM + ELM
(60)	IL	viscosity	ANN
(61)	DES	viscosity	MLR + ANN
(62)	cosmetic oils	viscosity	MLP
(63)	DES	viscosity, density	MLR
(64)	ester-alkane	mixing energy	ANN
(65)	DES	electrical conductivity	MLR
(66)	ES	density, viscosity	MLR
(67)	IL	viscosity, conductivity, density	SVR
(68)	ES	pH	MLR + ANN
(69)	DES	CO_2_ solubility	RF
(70)	chemicals	Abraham parameters, solvation free energy, solvation enthalpy	DNN
(71)	chemicals	molar mass, boiling temperature, vapor pressure, density, refractive index, aqueous solubility	DNN
(43)	F-refrigerants	vapor pressure	ANN
(72)	DES	pH	MLR + PLR + ANN
(73)	DES	surface tension	ANN
(74)	DES	electrical conductivity	ANN
(75)	IL + DES	infinite dilution activity coefficients	FM + DNN
(76)	polymers	glass transition, melting temperature	ANN
(77)	DES	eutectic composition, melting temperature	DT + MLR
(78)	IL	Henry’s law constants	SVM + RF + MLP
(79)	IL + DES	thermal conductivity	ANN
(80)	IL	surface tension, speed of sound	MLR + GBT
(81)	DES	CO_2_ solubility	ANN
(82)	DES	heat capacity	MNLR + ANN

a IL, ionic liquids; DES, deep eutectic solvents; MLR, multiple linear regression; RBNN, radial basis neural network; NN, neural network; MLP, multi-layer perceptron; ANN, artificial neural network; SVM, support vector machine; ELM, extreme learning machine; QSPR, quantitative structure–property relationship; RF, random forest; DNN, deep neural network; PLR, piecewise linear regression; FM, factorization machine; DT, decision trees; GBT, gradient boosting tree; MNLR, multiple nonlinear regression.

In the present work, we aim to develop an ANN model to characterize the flammable behavior of novel combinations of refrigerants, exploring what is consensually considered as “mixtures of tomorrow” within the HVAC (Heating, Ventilation, and Air Conditioning) domain. The model employs molecular descriptors obtained from the COSMO model for real solvents (COSMO-RS)^44,45^ as inputs to effectively correlate molecular characteristics with flammability of pure refrigerants and their blends. First, the developed ANN model is trained using an expansive data set composed of flammability of pure refrigerants and their binary and ternary blends, along with ANN configuration optimization based on statistical indicators. The optimized ANN configuration is tested by using flammability for quaternary blends as a demonstration of the predictive power of the model. Lastly, the ANN model is used in a fully predictive manner to assess the flammability of novel ternary blends comprised of CO_2_, hydrocarbons, and F-based refrigerants as tangible alternatives to the 2010s refrigerants, such as R410A and R134a, known for their environmental drawbacks.^46,47^ By synthesizing market needs, environmental concerns, and safety requirements, our approach intends to offer a pragmatic alternative that aligns with the urgent demands of today. Through this comprehensive endeavor, we hope to contribute to solving a complex problem by providing a meaningful solution, reinforcing the synergy between technological innovation and social responsibility.

## Methodology

**Figure 1:**
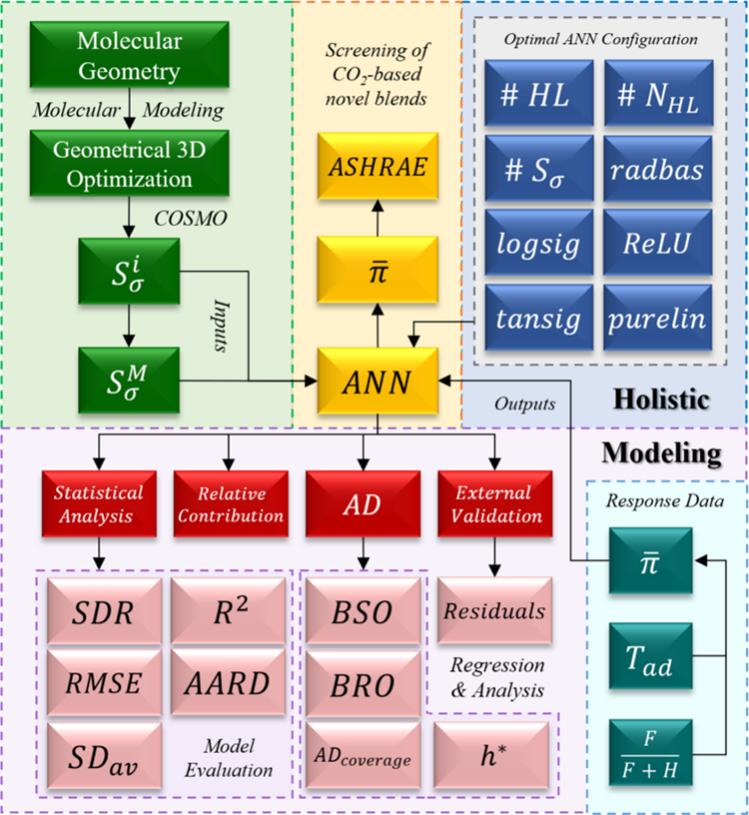
Integrated QSPR modeling framework implemented in this contribution.

The methodology proposed in this work, depicted in Figure 1, encompasses four-stages: first, we use COSMO-RS for data set generation in addition to supplying the essential output data for ANN training and subsequent fitting. This is followed by the second stage with the fine-tuning of the model’s inner layout, adjusting layers, neurons, and hidden layer activation functions for optimal performance. This step establishes a correlation between the COSMO-RS molecular descriptors (inputs) and the observed NFI outputs. The third stage entails a rigorous evaluation of the ANN, combining regression and statistical assessments with the external validation of refrigerant blends not included in the training stage. Upon concluding the model’s comprehensive testing and validation, including outlier detection, we proceed to the screening of industry-targeted future refrigerants in stage four. Here, the ANN is tasked with predicting the flammability characteristics of new, untested CO_2_-based mixtures, specifically in terms of ASHRAE flammability ratings.

### Flammability Output Data Set Assembly

The data set for ANN development comprises the flammability using NFI (π̅) as an output, for a comprehensive array of pure refrigerants, binary, and ternary blends compiled from multiple sources.^24,30,37,39,83^ Specifically, NFI values for 18 pure-components, 1309 binary mixtures, and 1028 ternary mixtures were extracted from Bell’s et al. contribution.^37^ Additionally, Calleja-Anta^39^ contributes one additional pure compound (R1132a), 8 binary mixtures, and 579 ternary mixtures. Further expansion of the data set stems from Domanski,^83^ which supplied a set of binaries, and Linteris et al.,^24,30^ introducing R161 and 180 novel binaries. The flammability of these refrigerants is represented through the NFI,^24^ which is an empirical representation for the flammability of working fluids estimated using the refrigerant’s adiabatic flame temperature (*T*_ad_), and degree of fluorination, expressed as the ratio of fluorine atoms to the total number of fluorine and hydrogen atoms . These parameters are fine-tuned to calculate the NFI according to eq 1. The temperature difference in the numerator is standardized by the upper limit adiabatic flame temperature of 2500 K. The *atan*2 function is employed to derive the four-quadrant arctangent angle within the domain [−π, π], accounting for coordinates in a two-dimensional Cartesian plane relative to the positive *x*-axis, prior to adjusting the perspective to span angles from −180 to 180°. The 1/2L boundary (π_1,2L_) is set at 36, yielding a normalized flammability index ranging from zero, at this threshold, to an absolute value of 100, for highly flammable compounds. Indeed, the flammability class for refrigerants in line with the ASHRAE classification are grouped based on their NFI values, with nonflammable class 1 (NFI ≤ 0), mild-flammable class 2L (0 < NFI < 50), and flammable classes 2 and 3 (NFI ≥ 50).
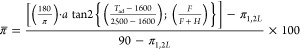
1

The NFI flammability output data set consists of a total of 3127 refrigerants including 20 pure refrigerants with eight hydrofluorocarbons (HFCs), five hydrofluoroolefins (HFOs), six saturated hydrocarbons (sHCs), and one additional hydroolefin (HO), along with 1500 binary blends, and 1607 ternary blends, with details on their distribution included in Figure S1 in the Supporting Information (SI). The data set for binary blends is predominantly weighted toward the lower end of the NFI, with 83.27% of data points falling within categories 1 (NFI ≤ 0), and 2L (0 < NFI < 50), featuring mostly binary blends of HFC + HFC and HFC + HFO (813 points), followed by combinations of HFO + HFO (144 points), HFC + CO_2_ (111 points), HFO + CO_2_ (91 points), HFC + sHC (74 points), sHC + sHC (30 points), and HFO + sHC (29 points). In contrast, the ternary blends flammability data set provides a lower representation of 1–2L blends, specifically comprising 394 data points for CO_2_-based blends, while also having higher representation of ternary blends in flammable classes of 2 and 3 (NFI ≥ 50), with 607 data points. This distribution underscores a well-balanced data set, aiding in the identification of safe refrigerants. It is noteworthy that lower NFI values are frequently correlated with CO_2_-based binary blends containing quantitatively small contents of other refrigerants, whereas NFI values of 100 occur in HCs-rich blends.

These data are used for ANN model development involving training, testing, and validation, while NFI for 55 quaternary blends are used for external validation by testing the developed ANN model on these unseen data.

### Input σ-Profiles Molecular Descriptors for Refrigerants via COSMO-RS

Toward predicting flammability of refrigerants, pures and blends, COSMO-RS is used to obtain molecular descriptors representative of the molecular structure and energy of the studied refrigerants, as ANN inputs. These descriptors are based on the σ-profile, which is the probability of specific charge density (σ) on a discrete surface segment, obtained from the density functional theory (DFT) level geometric optimization for molecules using COSMO-RS.^44,45^ Given the thorough coverage of functional groups by COSMO-RS, additional descriptors based on group contribution methods^84,85^ would increase the complexity of the ANN architecture without yielding significant enhancements in accuracy and predictive capability. While descriptors based on physical properties such as heat of combustion, flammability levels, critical points, and molecular weights, among others, hold potential for enhancing the modeling framework, their use is limited by the incomplete availability of data for the selected output assembly under consideration. This underscores the significance of this work, as it establishes a direct correlation between molecular characteristics and flammability, working with accessible inputs rather than depending on costly and resource-intensive experimental designs, which are often challenging for novel fourth generation systems.

The methodology for obtaining σ-profiles^86^ starts with importing the SMILES notation for each pure refrigerant into the Turbomole software (TmoleX v4.5.1).^87^ The three-dimensional (3D) molecular structures were subsequently refined through a geometrical optimization at the DFT level using the def-TZVP basis set with the Becke–Perdew 86 (BP86) generalized gradient approximation, and a rigorous self-consistent field (SCF) convergence criterion was set at 1 × 10^–6^ hartree.^43^ The optimized 3D molecular structures (exported as COSMO files)^88^ were transferred to COSMO-RS software (COSMOThermX v19.0.5)^89^ to generate the σ-profiles, each encompassing 61 data points in the σ-range of ±0.03 e/Å^2^, which in essence represent the polar and nonpolar regions across the molecule’s surface. The generated σ-profiles were then discretized into 61 regions, each with a screening charge density of 0.00098 e/Å^2^, used to compute the molecular descriptors selected as inputs for the ANN model, namely, *S*_σ-profile_, obtained as integrals of the area under the σ-profile curves in those 61 regions.^51^ To address computational demands while maintaining analytical robustness, an additional approach was considered for subsequent analyses: its truncation to an eight-term descriptor set.^68^ In this regard, each σ-profile was discretized into eight predefined electrostatic ranges representing the molecule’s surface polarity, and the numerical integral of each segment was computed using the integral function within MATLAB R2023a, as expressed in eq 2. This resulted in eight quantifiable descriptors that serve as reduced, yet meaningful, dimensional representations of the σ-profiles.
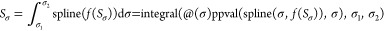
2where *S*_*x*_ serves as a truncated descriptor indexed from 1 to 8, each numerically capturing a specific electrostatic range within the σ-profile (*f*(*S*_σ_)), and essentially quantifying the area under the polynomic curve for that specific segment of interpolation. In this context, *S*_1_ focuses on σ/e·A^–2^ ranges from −0.030 to −0.0225, *S*_2_ from −0.0225 to −0.015, *S*_3_ from −0.015 to −0.0075, *S*_4_ from −0.0075 to 0, *S*_5_ from 0 to 0.0075, *S*_6_ from 0.0075 to 0.015, *S*_7_ from 0.015 to 0.0225, and *S*_8_ from 0.0225 to 0.03. The advantage of using these molecular descriptors as inputs is that, aside from being obtained *a priori* without fitting, they also contain sufficient information indicative of the structural and energetic nature of the molecules needed to predict their governing intermolecular interactions.

In the same manner, the molecular descriptors for binary and ternary refrigerant blends were obtained relying on the additive nature of the blend constituents’ σ-profiles.^61^ The σ-profile of a given blend is obtained as a linear combination of the constituent descriptors (*S*_σ_^*i*^), each weighted by their respective contributions, corresponding to their mole fractions (*x*_*i*_) in the blend, as
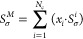
3

### Artificial Neural Networks

Although a variety of machine learning algorithms^90,91^ can be used to predict flammability of refrigerants using their molecular descriptors, artificial neural networks (ANN) are selected as the computational framework of choice not only for its exceptional accuracy but also due to its computational efficiency and architectural adaptability.^76^

ANNs draw their inspiration from biological neural networks and have undergone decades of evolution.^92,93^ While initially a tool to mimic biological intelligence, the focus has shifted toward their utility in solving complex engineering challenges, particularly in the domains of product design and safety assurance. The basic architecture of a feed-forward ANN^94^ comprises an input layer, one or multiple hidden layers, and an output layer. Each layer contains a varying number of neurons or nodes, and each neuron in the network is associated with a weight, a bias term, and an activation function that transforms the neuron’s output. Hidden layers perform transformations on input vectors through a series of linear and nonlinear operations to facilitate the mapping from the input feature space to the output target space, thereby enabling the approximation of complex, multidimensional functions. In this manner, the forward propagation of input data through the network is enabled via synaptic connections between neurons across successive layers. These connections, parametrized by weights and biases, serve as conduits for the computational flow, thereby facilitating the network’s ability to learn and model complex relationships between the input feature space and the output target.

In our model, the input feature vectors are derived from σ-profile descriptors,^51^ which in turn are obtained from quantum-level COSMO calculations, while the architecture of the feed-forward ANN is meticulously crafted using the advanced functionalities offered by MATLAB’s Neural Network Toolbox. The Levenberg–Marquardt (LM) algorithm integrated into MATLAB was utilized for its optimal balance of computational efficiency and stability in weight optimization for medium-sized data sets,^95^ using mean squared error (MSE) as the loss function, and initializing weights randomly. Compared to other algorithms like second-order Broyden–Fletcher–Goldfarb–Shanno (BFGS), LM offers faster convergence rates, robustness against local minima, and efficient memory utilization, making it a versatile and reliable choice for our study’s specific needs. Training proceeded for up to 2000 iterations (also known as epochs), with early stopping criteria incorporated to mitigate overfitting.

The input/output data set underwent partitioning into three principal subsets—training, validation, and testing—via the application of the “cvpartition” algorithm.^96,97^ This division technique is designed to bolster the model’s predictive performance and generalizability.^98^ The allocation of data into these subsets occurs randomly yet in a stratified fashion, based on predefined user ratios (80% allocated to training, 20% to validation and testing, evenly split). By employing stratification,^99^ the algorithm maintains an analogous distribution of classes across the training and testing subsets, thereby ensuring that the testing subset accurately embodies the characteristics of the full data set. While randomization introduces an element of variability, enabling different outcomes across multiple runs, the replicability of results is assured through the “genfunction” utility introduced in the MATLAB coding.

Several configurational parameters for ANN development were tested to determine optimal ANN structure including the number of neurons in each hidden layer (1 up to 25 neurons) and hidden layer activation function (appreciate mathematical expressions in Table S1) such as hyperbolic tangent, logistic sigmoid, linear, radial basis, and rectified linear unit, selecting the final architecture of our ANN based on optimal statistical indicators.

### ANN Model Evaluation

The developed ANN model is subject to a rigorous evaluation via an assortment of statistical metrics in order to determine the accuracy of its training and testing processes.^66,81,100^ Specifically, we relied on the coefficient of determination (*R*^2^), root-mean-square error (RMSE), average absolute relative deviation (AARD), and average standard deviation (SD_av_), given in eqs 4–7, where *N* is the number of observations, while  and  are the mean of the actual and predicted values. The best performing model would possess a high *R*^2^ value close to unity, and low RMSE, AARD, and SD_av_ values.
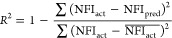
4
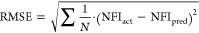
5
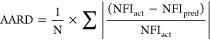
6
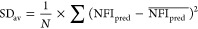
7

#### Applicability Domain

In order to determine the predictive power of the developed ANN model, particularly extrapolative capabilities, the applicability domain (AD) is used,^101−104^ working as an outlier detection mechanism, while also demarcating the space in which the model’s predictions can be considered scientifically reliable, thereby establishing a domain for extrapolations.

To build this multidimensional space, leverage values (*h*_*i*_) and standardized residuals (SDR) are integrated and visualized via William’s plots.^105^ Leverage values are determined by eq 8, where *z*_*i*_ corresponds to the descriptor row vector for molecule *i*, and *Z* to the descriptor matrix associated with the training set, essentially assessing a molecule’s similarity to the data set’s central tendency. Concurrently, SDR evaluates the model’s predictive capability using eq 9, where σ^2^ is the residual variance. Furthermore, it is essential to introduce a warning leverage threshold, h*, which serves as an upper limit to flag predictions that are less trustworthy due to a higher degree of extrapolation. This is calculated using eq 10, incorporating d*, the count of descriptors, and *p*, the total number of samples in the training set. Operational boundaries in the William plot are set when accounting a SDR checkpoint of ±3 units, instrumental for quantitatively assessing the AD’s coverage (see eq 11), where *p*_AD_ represents the number of data points within the AD. Combined, these metrics and visual tools contribute to a holistic comprehension of the QSPR model’s operational scope, aiding in both validation and subsequent application.
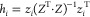
8
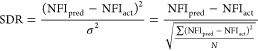
9
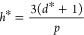
10
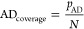
11

#### Input Relative Contribution Assessment

In the pursuit of understanding the intricate relationship between input descriptors and their significance of predicting output response in the context of neural networks and regression analysis, we employ an analytical approach known as the partial derivatives (PaD) method.^106−108^ At the heart of this approach is the concept of differentiation, which essentially measures the rate of change of a function’s output relative to infinitesimal alterations or perturbations in its input variables.^104^ This is achieved by computing the partial derivative of the output with respect to each individual input descriptor, offering insights into the significance or relative contribution of each descriptor to the system’s response. In this study, we employed the PaD method via the limit approach, using a Δ*S*_σ_ value of 0.5% (0.0001% when *S*_σ_ = 0) for approximation, enabling a precise examination of infinitesimal variations and deepening insights into the system’s intrinsic dynamics. Using eq 12, the magnitudes of the partial derivatives indicate the dynamic effect of each input on the assessed output, highlighting their relative contribution. Upon determination of all data points, a matrix space is structured, as in eq 13, wherein the summation of given columns denotes the relative influence of the corresponding input on the assessed response.
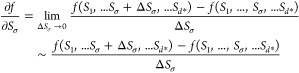
12
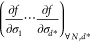
13

## Results and Discussion

### σ-Profiles of Pure Refrigerants from COSMO-RS

**Figure 2:**
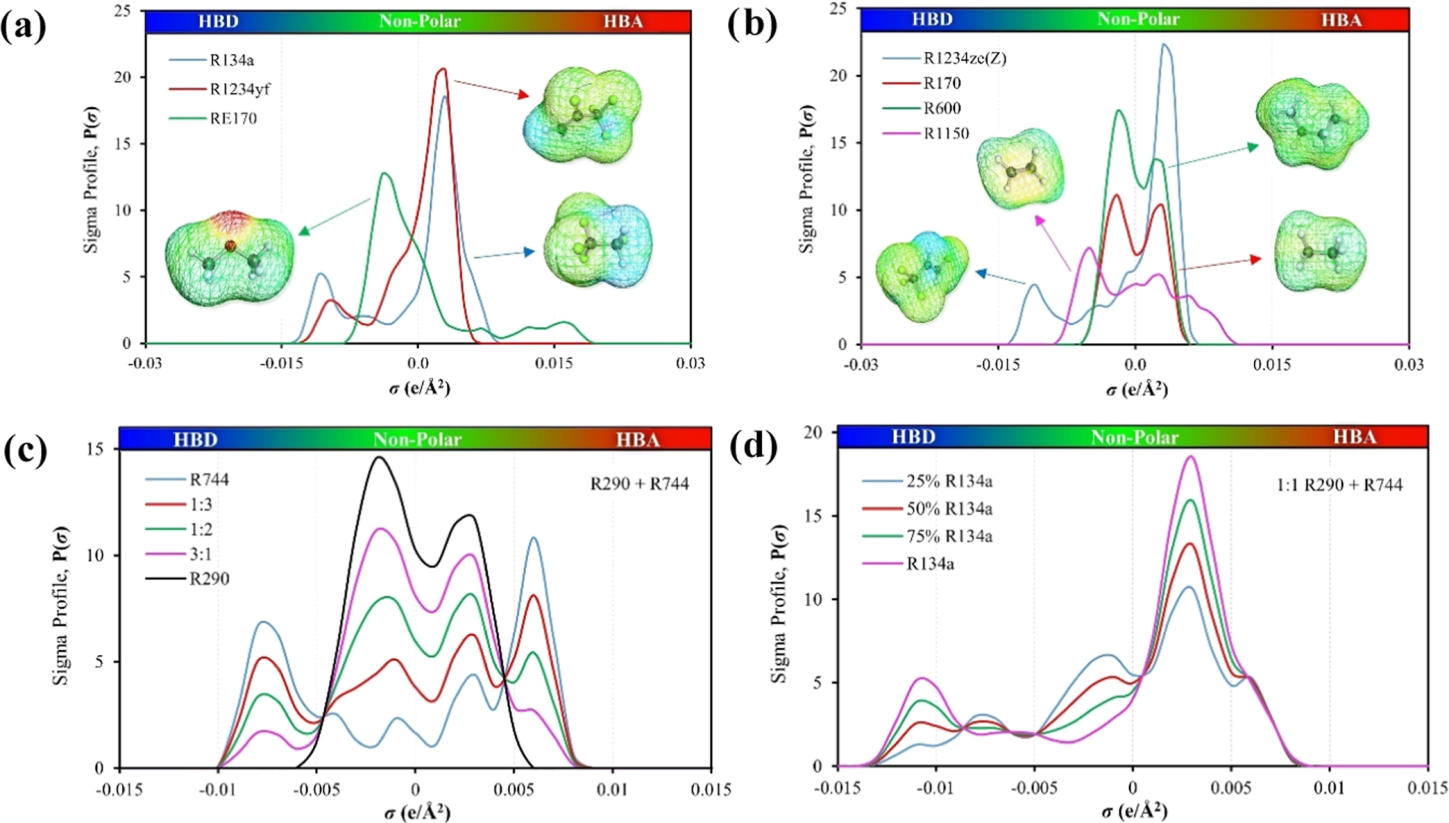
σ-Profiles of (a, b) selected single-component refrigerants used in this work, (c) R744 (blue) + R290 (black) binary mixtures at 25 (red), 50 (green), and 75 (pink) mole% ratios of R290, and (d) R744 + R290 at 1:1 ratio with the addition of 25 (blue), 50 (red), 75 (green), and 100% (pink) of R134a mole fractions.

In this work, we evaluated the σ-profiles of 23 refrigerants listed in the NFI database. This included 20 pure-components (see section Methodology for extended details), as well as R1270, CO_2_, and RE170 used in binary and ternary combinations, highlighted in Figures 2 and S2, providing detailed insight into their governing interactions and their role on their flammability. This concept relies on evidence that COSMO-RS descriptors effectively capture factors influencing flammability by considering surface charge density and molecular interactions, including the presence of specific functional groups, polarity, oxygen content, hydrogen bonding capacity, and the presence of heteroatoms such as fluorine or chlorine.

For instance, in the case of HFC 1,1,1,2-tetrafluoroethane (R134a), in Figure 2a, the highly electronegative fluorine atoms lead to a pronounced electron density difference in the C–F bonds, making them considerably polar. These zones of high interaction potential would correspond to the peaks in the hydrogen bond donor (HBD) region of the σ-profile.

Similarly, the HFO 2,3,3,3-Tetrafluoropropene (R1234yf), in Figure 2a, also displays areas of high polar interaction potential due to its C–F bonds, but additionally possesses C=C bonds, which present another unique reactivity zone on the molecule’s surface. Note that the C–F bond is highly polar due to the significant difference in electronegativity between carbon and fluorine. This bond is a strong hydrogen bond acceptor (HBA) and would typically be represented in the σ-profile with a high surface charge density (positive σ value). The C=C bond, instead, has π-electrons that can act as a weak HBA and would be represented in the σ-profile in a region with a moderate surface charge density, as it is less polar than the C–F bond but more polar than nonpolar bonds such as C–C or C–H. The σ-profile for the HFO trans-1,3,3,3-tetrafluoroprop-1-ene (R1234ze(E)), in Figure S2, with fluorine atoms on opposite sides of the bond, often exhibits moderately lower peaks due to a less polar configuration. Conversely, a different spatial arrangement around the C=C bond as with its isomer cis-1,3,3,3-tetrafluoroprop-1-ene (R1234ze(Z)), see Figure 2b, displays higher peaks, reflecting the stronger polar environment. These peak disparities indicate differing atomic contributions to each isomer’s overall molecular polarity.

In contrast, carbon dioxide (R744), in Figure 2c, though nonpolar overall, has zones of significant electron density due to its polar covalent C=O bonds. Such polar character results in a different σ-profile with polarity zones associated with the oxygen atoms. In a similar manner, the oxygen atoms in dimethyl ether (RE170), in Figure 2a, induce zones of high surface charge density due to their high electronegativity, indicating strong HBA characteristics. However, the presence of fluorine atoms and a C=C bond adds complexity to its σ-profile, with higher peaks from the C–F bonds (strong HBA) and moderate peaks from the C=C bond (weak HBA). Hydrocarbons such as ethane (R290), in Figure 2c, exhibit a σ-profile dominated by lower σ values, indicating nonpolar regions primarily due to C–H and C–C bonds, thus reflecting hydrocarbons’ overall nonpolar nature.

In binary mixtures of CO_2_ with R290 (see Figure 2c), a gradual shift toward R290's nonpolar profile is observed, characterized by the diminishing HBD/HBA interactions of CO_2_. This effect underlines a direct correlation between molecular interactions, as revealed by sigma profiles, and the mixture’s overall flammability, emphasizing the significance of molecular composition in determining refrigerant behavior and safety. Further complexity arises in the ternary mixture of R290 and CO_2_ with the inclusion of R134a at equimolar ratios, as depicted in Figure 2d. As the fraction of R134a in the ternary mixture increases, there is a marked decrease in flammability, partially marked by a transitional enhancement in the nonpolar peak around 0.0025 e/Å^2^ of the sigma profile, accompanied by a modest increase near the HBD region.

### Selection of the Best Optimal ANN Configuration

As previously highlighted, the ANN for predicting the NFI for pure refrigerants and blends relying on their molecular descriptors from COSMO-RS has a two hidden layer general architecture. According to empirical evidence from various machine learning models,^43,73,76,79,81^ this particular setup constitutes a suitably deep network, optimally balancing learning capacity, computational efficiency, and generalization performance, thereby mitigating the risk of overfitting. The number of neurons in each hidden layer and activation functions were kept as variables to determine the best ANN configuration.

**Figure 3:**
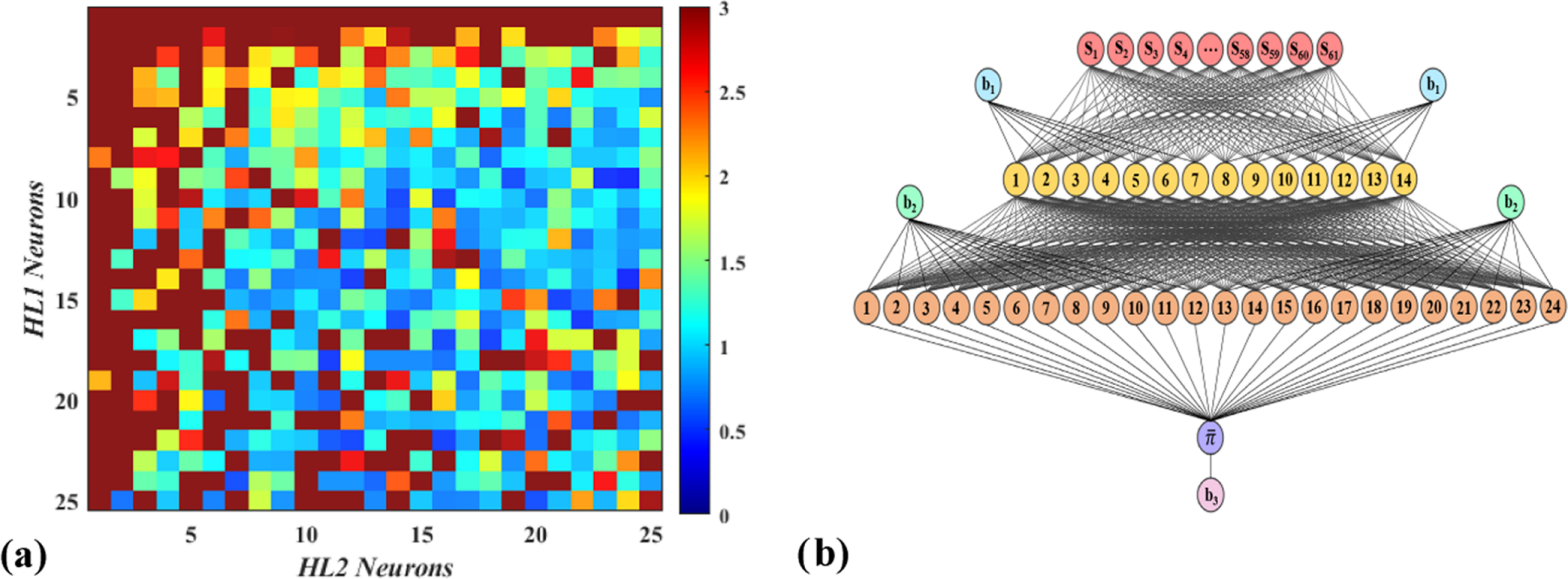
(a) Mapping the average RMSE variation in relation to the number of neurons in first (HL1) and second hidden layers (HL2) while using tansig activation function in both HL1 and HL2, and (b) The configuration for the best performing ANN of [61 (I) × 14 (HL1) × 24 (HL2) × 1 (O)] to predict the NFI of refrigerants.

First, the number of neurons in each of the 2 hidden layers were changed from 1 to 25 neurons, running each network for eight randomized trials for each configuration, with their average RMSE as a function of the number of neurons in each hidden layer shown in Figure 3a. The most effective configuration is found to be a [61 (I) × 14 (HL1) × 24 (HL2) × 1 (O)] configuration shown in Figure 3b, with the lowest average RMSE of 0.430. This configuration ensured a RMSE < 1 in each of its randomized trials, signifying high precision and accuracy in all of its random tests. Note that this ANN configuration requires a total of 1190 weights and 39 biases to effectively correlate the molecular descriptors with the NFI of the refrigerants.

Additionally, for ANN configuration optimization, we can observe that arrangements with only a single neuron in any of the hidden layers often correspond to higher error rates, thereby highlighting the limitations of overly simplistic models. However, the process is not as straightforward as merely maximizing neuronal count; a configuration involving a high number of neurons in both hidden layers, such as [61 (I) × 25 (HL1) × 25 (HL2) × 1 (O)], can paradoxically lead to increased RMSE (i.e., RMSE = 2.91). This underlines the delicate balance between model complexity and performance in the context of the ANN architecture.

To further enhance the performance of the best ANN configuration depicted in Figure 3b, a series of additional modifications were carried out, such as (1) introducing a third hidden layer, (2) reducing the number of descriptors to 8 rather than the initial 61, and (3) changing the activation functions for the hidden layers. The introduction of an extra hidden layer sets the stage for a dichotomy: although it can potentially increase the model accuracy (i.e., reduce RMSE), this would be at the expense of increased computational time. Conversely, reducing the descriptor count is expected to reduce the computational time, yet potentially at the cost of a reduced accuracy (i.e., increased RMSE). Hence, our task is to adjust these variables, finding the right balance between reducing the computational time and minimizing the RMSE.

As provided in Figure S3a, adding a third hidden layer to the pre-established optimal configuration (i.e., [61 (I) × 14 (HL1) × 24 (HL2) × 1 (O)]) does not significantly lower the RMSE, regardless of the number of neurons in the third hidden layer (i.e., 1–25 neurons), only observing a slight decrease in RMSE when using either 4 or 6 neurons in the third layer. However, this comes at the expense of a 3-fold and 8-fold increase (i.e., ET/s = 18.9 for ANN configuration in Figure 3b) in the computational time (measured as the elapsed time per epoch), respectively, negating the minor enhancements in accuracy, thus keeping the configuration of the best performing model unchanged. The reduction of molecular descriptors from 61 to 8 is analyzed in Figure S3b. As expected, computational time substantially decreases from 0.703–0.803 s to 0.155–0.181 s when using the 8 descriptors. Nevertheless, this substantial decrease in computational time produces a severe deterioration of the ANN performance, leading to a substantial increase in the RMSE. The least error, achieved via [8 (I) × 24 (HL1) × 19 (HL2) × 1 (O)] configuration, exceeds an RMSE of 6.00, indicating a decrease in model accuracy. Comparatively (see Figure 3a), when using 61 descriptors, 86.4% of the total tested combinations culminate in a lower error value, typically with RMSE = 1. In summary, although the model performs calculations faster when using fewer descriptors, this speed comes with a cost in accuracy, compromising the practical application of the model, and accordingly, the number of inputs was kept at 61 descriptors.

Having identified the [61 (I) × 14 (HL1) × 24 (HL2) × 1 (O)] configuration as optimal, we have conducted a systematic examination of various activation functions within the hidden layers, including hyperbolic tangent (*tansig*), logistic sigmoid (*logsig*), linear (*purelin*), radial basis (*radbas*), and rectified linear unit (*ReLU*) functions, in order to fine-tune the performance for this specific configuration. The detailed results of this examination, averaged over ten random runs of the corresponding ANN configuration, are outlined in Table 2. From these observations, the *tansig* function is the best performing, achieving an *R*^2^ of 0.9997 and RMSE of 0.5847 across all data sets (i.e., training, testing, validation). The *logsig* function is another promising alternative on par with *tansig* function achieving an *R*^2^ of 0.9996 and RMSE of 0.6651. Its consistent performance across validation and testing data sets (*R*^2^ of 0.9990 and 0.9987, respectively) highlights its efficacy in predicting external data sets. The *ReLU* and *radbas* functions, although competent, exhibit lower *R*^2^ values compared to *tansig* and *logsig* functions, hinting at a potential overfitting. However, the most notable discrepancy is observed with the use of the *purelin* function with an overall *R*^2^ of 0.8984, denoting a considerable mismatch with the actual data.

**Table 2:** Analysis of the Performance for Activation Functions in the Hidden Layers of the ANN Model

function	equation	range	set	*R*^2^	RMSE	AARD/%	SD_av_
hyperbolic tangent (*tansig*)		[−1, 1]	train	0.9998	0.4299	3.9778	0.4268
			validate	0.9991	1.0437	4.3304	1.0434
			test	0.9994	0.8629	4.5404	0.8627
			**total**	0.9997	0.5847	4.0694	0.5827
logistic sigmoid (*logsig*)		[0, 1]	train	0.9998	0.4268	3.8884	0.4244
			validate	0.9990	1.1260	3.8893	1.1250
			test	0.9987	1.1822	6.1410	1.1800
			**total**	0.9996	0.6651	4.1140	0.6639
linear (*purelin*)		[−∞, ∞]	train	0.9011	11.3709	92.1066	11.3732
			validate	0.8988	11.5049	87.1327	11.4848
			test	0.8785	12.6966	76.6944	12.7037
			**total**	0.8984	11.5324	90.0660	11.5336
radial basis (*radbas*)		[0, 1] | *x* ∈ [0, ∞)	train	0.9691	5.5479	47.5140	5.5477
			validate	0.9425	7.8847	45.1713	7.8874
			test	0.9496	7.1821	57.1790	7.1870
			**total**	0.9643	6.0473	48.2469	6.0469
rectified linear unit (*ReLU*)		[0, ∞)	train	0.9982	1.4754	14.1345	1.4747
			validate	0.9948	2.4795	14.8493	2.4790
			test	0.9955	2.3021	17.6914	2.3022
			**total**	0.9976	1.7079	14.5621	1.7073

In light of the previous analysis, it becomes manifest that the optimal ANN for predicting NFI values for refrigerants using molecular descriptors from COSMO-RS is the [61 (I) × 14 (HL1) × 24 (HL2) × 1 (O)] configuration using *tansig* activation functions in both hidden layers. This synthesis of insights paves the way for us to explore this specific configuration further, considering it as the best-case scenario for our targeted application.

### Evaluation of the Best Optimal ANN Configuration

#### Training, Testing, and Validation of the Selected ANN Model

**Figure 4:**
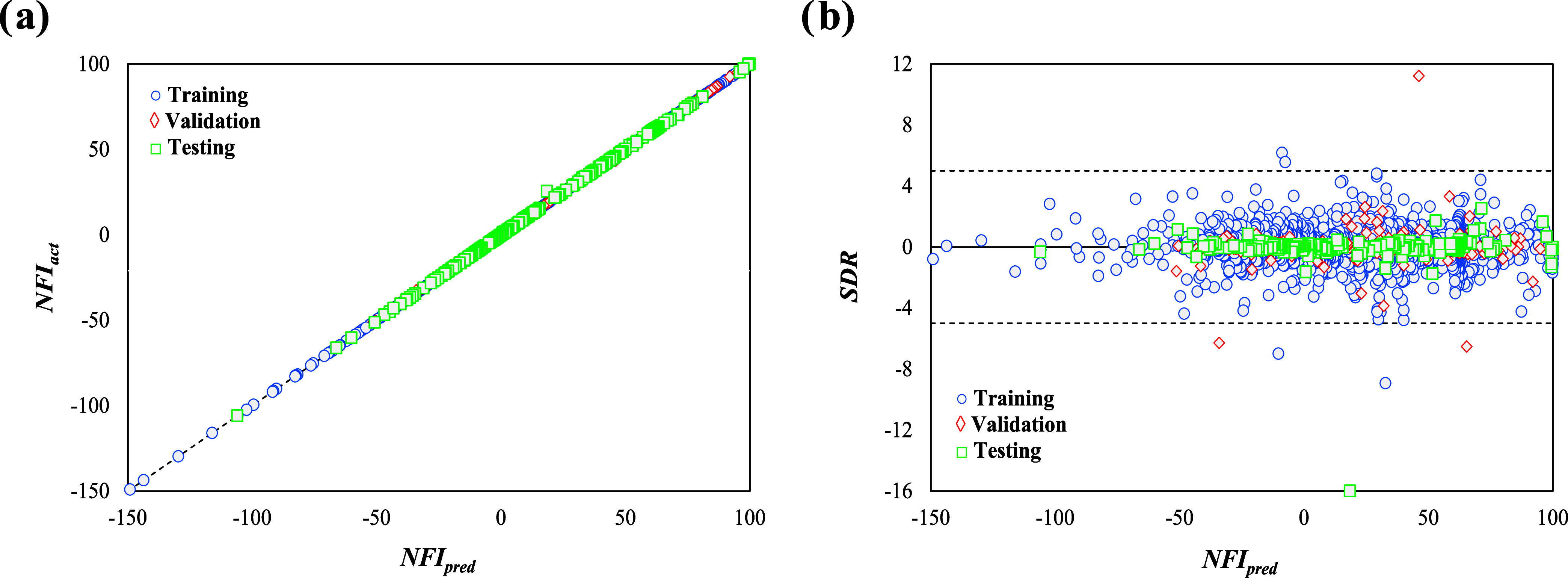
(a) Parity and (b) standardized residual plots between the actual and ANN-predicted NFI.

Figure 4a offers a visual comparison between actual and predicted NFI values for both training and external (validation and testing) sets, as a parity plot between ANN-predicted NFI (*x*-axis) and actual NFI data (*y*-axis) for the best performing ANN configuration from the previous section. The machine learning simulation achieved the desired accuracy at epoch 134 in just over a minute (00:01:03), with a performance error of 0.00491. It reported a gradient of 2.83, and a μ of 0.001, striking a balance between training strategies. These results confirm the successful training of the model, as most points fall along the *y* = *x* diagonal. Consequently, the model is seen as a reliable tool for predicting flammability based on the given inputs, effectively avoiding overfitting, a common pitfall in machine learning. This accurate fitting underscores the model’s precision and narrow dispersion in future predictions, as indicated by the excellent alignment with actual data in the external testing and validation data sets.

The residual plot, shown in Figure 4b, highlights the model’s ability to predict the flammability index for binary and ternary mixture blends. As observed, most of the differences between predicted and actual values (residuals) are within a range of ±1, while almost all fall within a broader range of ±5 SDR, with only a few exceptions. Specifically, 0.72% of the training data, 0.96% of the validation data, and 0.32% of the testing data fall outside the SDR range of ±5, while a substantial 81.3% of the total data set is found to be constrained within an SDR of ±1. The data analysis reveals that in the worst-case scenario within an SDR of ±5, the NFI is overestimated by 0.27; however, given that the NFI spans a substantial range from −150 to +100, this overestimation is considered minor and does not constitute a clear outlier. Certainly, the comprehensive coverage of all safety classifications within the flammability scope underscores the database distribution’s high reliability and accuracy. Notably, only two points (1.9% of the validation set) within the 2–3 flammability region, and a mere 0.44% of total validation data in the 1–2L discretized spectrum, display residual deviations over ±5, respectively, with no outliers detected during testing.

The comprehensive results of the statistical analysis, including specific key performance indicators, are presented in Table 3 to supplement the previous visual representation with statistical insights. Our findings indicate that the *R*^2^ value remained consistently high across all data sets, registering values greater than 0.999. Although demonstrating a robust linear relationship between predicted and actual NFI values, this consistency fails to delineate a clear trend; hence, further assessment through additional statistical descriptors becomes essential. Unlike *R*^2^, RMSE and AARD show a clear trend as the analysis moves from training to validation and testing stages, with values increasing progressively, thereby indicating the model’s increasing deviation as it is exposed to new and unseen data. Note that while RMSE quantifies aggregate differences between predicted and actual values, AARD measures the average percentage deviation from the actual values, providing a complementary view of the model’s performance across different stages of data analysis. This insight underscores the importance of careful evaluation in understanding how well the model may perform when applied to real-world scenarios. Lastly, the SD_av_, quantifying the residuals’ dispersion or spread around the mean, reflects the inherent variability and complexity of each data set, as the larger the deviation, the more the residuals spread out from the mean. This assessment reveals that the predictions for the validation and testing stages are more spread out from the average error, highlighting the importance of both the underlying data distribution in addition to the model’s capacity to handle diverse complexities and inherent variations within different data sets.

**Table 3:** Statistical Analysis of Performance Parameters for the Developed ANN Model

metric	training	validation	testing	total
*R*^2^	>0.999	>0.999	>0.999	>0.999
RMSE	0.0727	0.2546	0.4402	0.1735
AARD%	0.7302	0.7957	1.1989	0.8091
SD_av_	±0.0357	±0.0679	±0.0812	±0.0434
data points	2501	313	313	3127

#### Applicability Domain (AD)

**Figure 5:**
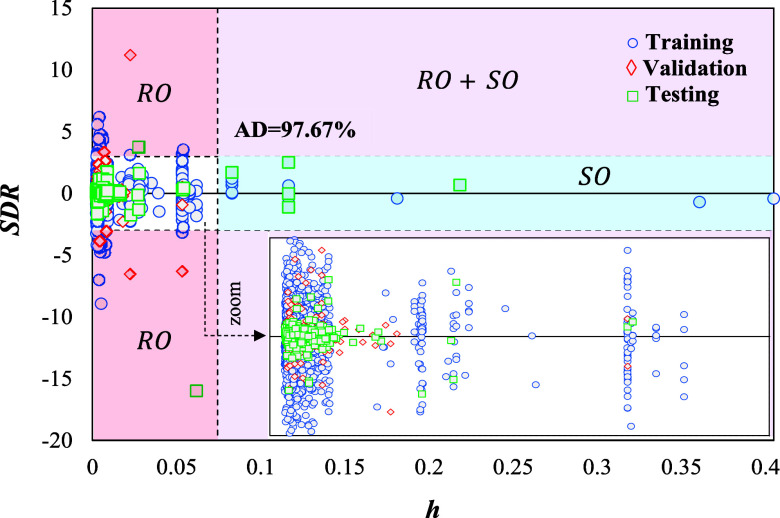
William’s plot delimitating the AD (white) boundaries for the total set of assessed compounds, with response outliers in red, structural outliers in turquoise, and areas with both in purple.

An examination of Figure 5 and Table 4 provides insights into the applicability domain of the developed ANN model, contributing to the identification of potential outliers and anomalous data points lacking a physical interpretation. Within this context, 20 structural outliers were identified, distributed in 15 and 5 from the training and testing stages, respectively. For assessing data points where *h_i_* values range between h* (0.0744) and 0.1, identified as borderline structural outliers, the number of instances falling outside the AD diminishes to 14, with 10 related to training and 4 to testing stages. In addition, the analysis reveals that response outliers, defined as data points with an SDR value outside ±3, tally up to 55, comprising 47 for training, 6 for validation, and 2 for testing stages within the developed ANN. If the borderline response outliers (SDR between ±5) are included within the AD, the outliers diminish to 8, distributed as 4 for training, 3 for validation, and 1 for testing. This observation revealing that most response outliers cluster near the SDR border of ±4, defining a clear threshold between potential interpolation and extrapolation predictions. Figure 5’s zoomed view reveals an average AD coverage of 97.67% across the total 3D space, with 97.92% of the data points for external model evaluation falling within the AD. This percentage rises to 99.36% when including borderline outliers for both structural and response factors, reflecting the trend that most response outliers converge at the SDR border’s limit. The cumulative evidence, as portrayed through the visual and statistical elements of Figure 5 and Table 4, underscores the robust alignment of the ANN model, establishing that new predictions within this domain can be deemed to be reliably consistent for comprehensive analyses and high-throughput screening applications.

**Table 4:** AD Parameters, Including Borderline Outliers in Parentheses, for the Developed ANN Model^a^

	training	validation	testing	total
structural outliers (BSO)	15 (5)	0 (0)	5 (1)	20 (6)
response outliers (BRO)	47 (43)	6 (3)	2 (1)	55 (47)
AD_coverage_ (+ BO)/%	97.60 (99.52)	98.08 (99.04)	97.76 (98.40)	97.67 (99.36)

a h* = 0.0744.

#### Relative Contributions and Importance of Input Parameters

**Figure 6:**
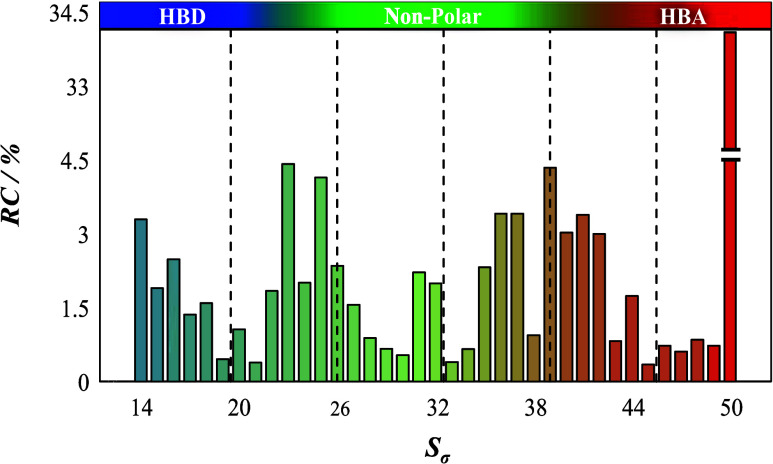
Relative contribution of the input parameters used in the ANN model.

Figure 6 illustrates the relative contribution of the 61-descriptor inputs to the NFI output, where the greater the absolute value of the contribution, the more pronounced the discrimination capability of the respective parameters. From the analysis, it becomes apparent that the primary contributor to the NFI is S_50_ (, @σ = 0.019 e/Å^2^), accounting for 34.1% of the total contribution. This is followed by the notable influences of *S*_23_ (σ = −0.008 e/Å^2^), *S*_39_ (σ = 0.008 e/Å^2^), and *S*_25_ (σ = −0.006 e/Å^2^), contributing 4.42, 4.24, and 4.14%, respectively. Further emphasizing the importance of certain regions, descriptors *S*_14–18_, *S*_20_, *S*_22–27_, *S*_31_, *S*_32_, *S*_35–37_, *S*_39–42_, *S*_44_, and *S*_50_ account for a remarkable 90.98% of the total contribution. Certainly, it is evident that polarized positive-charged segments (depicted in intense blue) and negative-charged segments (in intense red) significantly impact the NFI of the refrigerant mixtures, surpassing the weak hydrogen acceptor and donor regions, with the nonpolar zone emerging as the least significant factor. This observed trend, evident from the data, aligns with the interplay of hydrogen bonding capabilities and electron-donating and -accepting characteristics within the molecular structure. In this context, mildly strong acceptor or donor regions significantly influence the NFI through stable and potent interactions, while weak donors, acceptors, and nonpolar regions yield lesser or minimal effects, due to their inherently weaker interactions that lack substantial influence on flammability dynamics.

In a further examination of the underlying dynamics, it is revealed that the molecular descriptors corresponding to *S*_1–13_ and *S*_51–61_, regions typically characterized by their role as predominantly HBD and HBA, respectively, manifest a complete absence of contributions. A plausible hypothesis for this observation might be rooted in the formation of stabilizing hydrogen bond networks within the molecular structure. These networks could act to stabilize the molecular system, reducing the available reactive sites and, thereby, diminishing the propensity for ignition. The intricate charge distribution and potential influence of the surrounding molecular features might further mitigate the contributions of these charged regions to flammability. To sum up, the insights gathered from Figure 6 not only underline the vital descriptors influencing the ignition features but also provide a deeper understanding of the underlying molecular interactions, which are central to the design and optimization of refrigerant mixtures.

### Testing ANN Predictive Power on Flammability of Quaternary Mixtures

As illustrated in Figure 6, a substantial 90.98% of the total contribution to the NFI is ascribed to a specific set of 23 molecular descriptors. The next step of the analysis involves a 2-fold approach encompassing both a comprehensive examination employing the full array of 61 molecular descriptors and a targeted assessment utilizing the condensed subset of 23 specific descriptors. Note both strategies employ consistent hidden layer layouts and activation functions, as detailed in the main outputs of Figures 3 and S3 and Table 2. The ANN is rechecked by employing 55 quaternary mixtures not previously used for training or testing the model, involving CO_2_, HFOs (R1243zf, R1234yf, and R1234ze(E)), and HFCs (R41, R134a, R227ea, R125, R32, and R152a), representing a wide spectrum of compositions. Specifically, the ANN is exclusively trained and validated on pure substances and binary and ternary mixtures (see data assembly and preprocessing section), rendering these quaternary mixture predictions as a stringent test of model integrity. For this purpose, the ANN configuration in Figure 3b operates using weights, biases, and output parameters as determined from Table S1, in accordance with the mathematical expression presented in eq 14. Building on this configuration, the ensemble neural model is integrated into an Excel spreadsheet in the Supporting Information, enabling NFI calculations across pure to quaternary refrigerant mixtures. Also, the reader may find the database for training and testing, in addition to the quaternary compositions used for external validation, as outlined in the “DataBase” and “External Validation DataBase” subtabs.
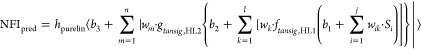
14

**Figure 7:**
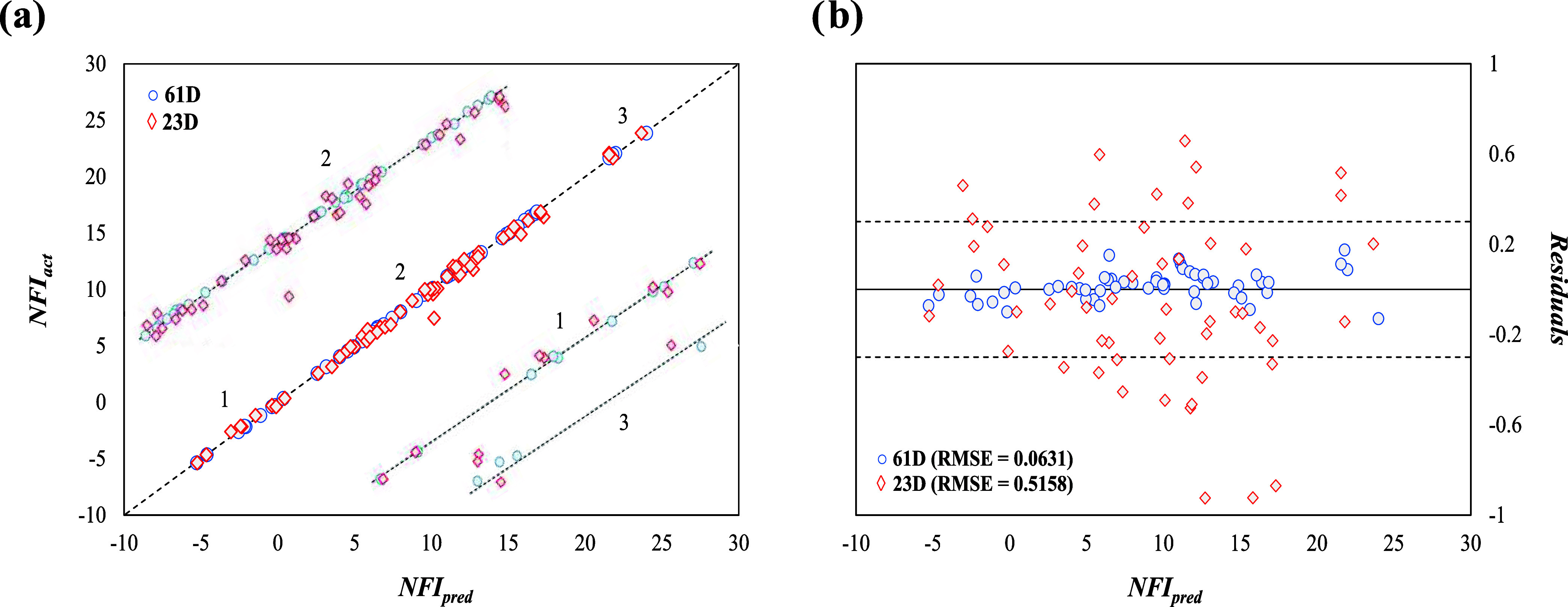
(a) Parity plot (numbers denote the flammability region zooms) and (b) residuals plot of actual^37^ versus ANN-predicted NFI values for selected quaternary mixtures using the 61 (blue symbols) and 23-descriptor (red symbols) ANN models.

As can be extracted from Figure 7, the 61-descriptor model achieves an RMSE of 0.0631, indicative of high accuracy, whereas the 23-descriptor model provides a less precise RMSE of 0.5158 but offers the advantage of reduced computational time. As appreciated, the increase in the RMSE represents a decrease in prediction accuracy, reflecting once again the complexity of capturing intricate molecular interactions with a reduced descriptor set. Figure 7 presents an in-depth and detailed graphical analysis of these trends. Specifically, Figure 7a contrasts the actual and predicted NFI values using both the 61 and 23-descriptor sets, while Figure 7b showcases the distribution of residuals, revealing that 41.8% of the total data exhibit a residual of ±3 when employing the simplified 23-descriptor model, an absent phenomenon in the data analysis concerning 61 descriptors. Interestingly, the AD method’s application disclosed that only one mixture, composed of R1243zf, R1234ze(E), R1234yf, and CO_2_ (52:32:12:4 mol %), falls outside the AD when using 23 descriptors, whereas a 100% AD is achieved with 61 descriptors. In summary, the 61-descriptor approach emerges as highly predictive (average residual of 0.049) but computationally more demanding, while the more simplified model, although less precise with an average residual of 0.34, offers the benefit of reduced computational time and complexity. Given the broad NFI prediction range evaluated [−5 to 25], the excellence of the ANN model’s predictive capabilities, particularly with 61 descriptors, stands as an impressive achievement.

### Predicting Flammability of Novel Ternary Refrigerant Blends

After a robust phase of training and testing, including external validation with quaternary mixtures, the ANN model is deployed to predict the ignition propensity of novel ternary mixtures of high interest for which no experimental data are available, marking their novelty in industry as a focal objective of this research. This crucial undertaking is instrumental in facilitating the selection of refrigerant blends that strike an optimal balance between a low GWP and moderate to low flammability without compromising safety-related characteristics.

**Figure 8:**
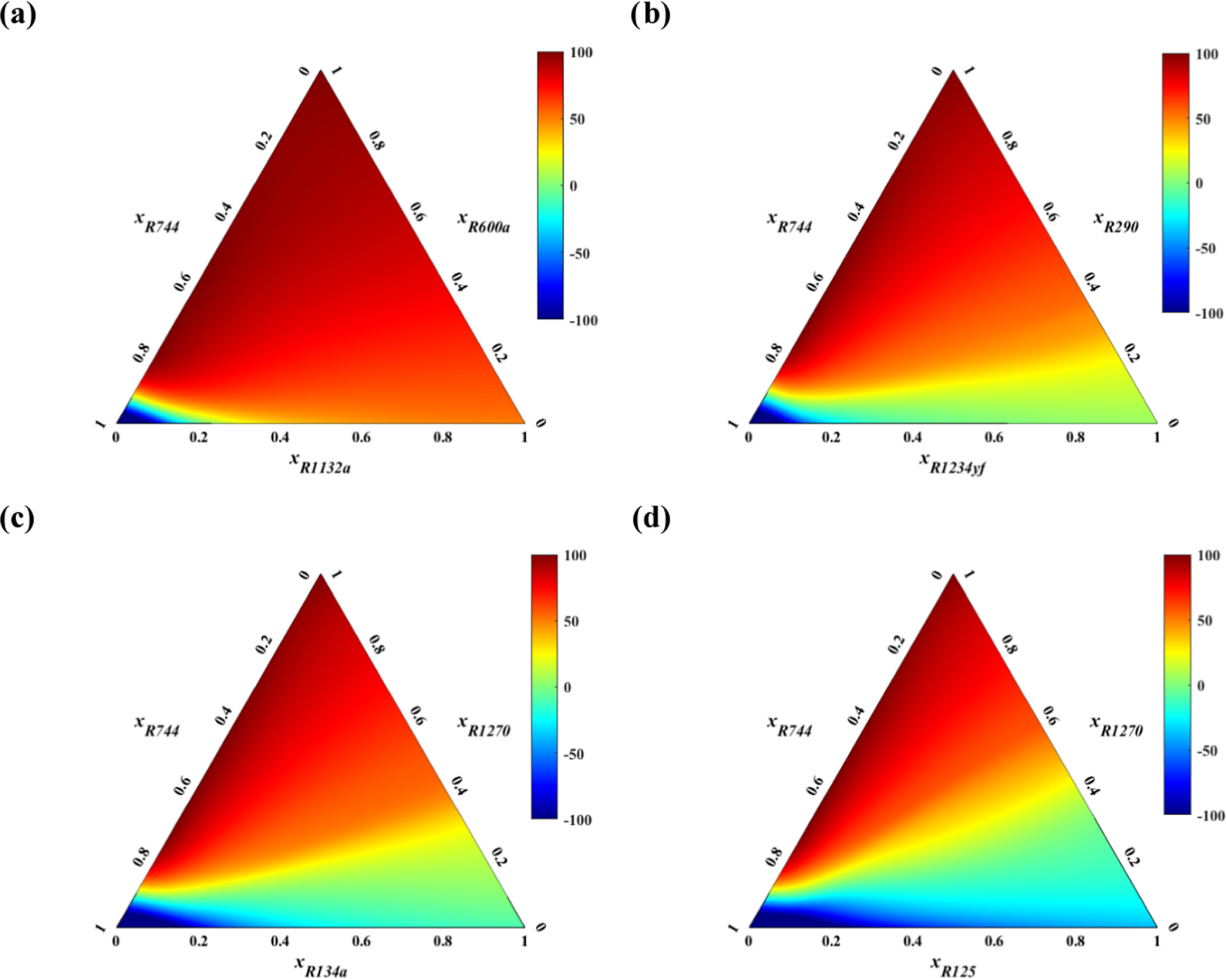
Ternary contour plots generated by multitask ANN, depicting predictions of NFI for various CO_2_ blends, including (a) R600a + R1132a, (b) R290 + R1234yf, (c) R1270 + R134a, and (d) R1270 + R125.

Figure 8 depicts composition-property correlation mappings of four specific mixtures, including (a) CO_2_, R600a, R1132a; (b) CO_2_, R290, R1234yf; (c) CO_2_, R1270, R134a; and (d) CO_2_, R1270, R125. This graphical representation serves as a comprehensive visual guide, elucidating the interplay among pure compounds at the vertices and binary mixtures along the edges. As observed, all formulated mixtures incorporate CO_2_ as a consistent component, complemented by commercialized carbon-based specimens R600a, R1270, or R290, and paired with F-based refrigerants with flammability ranging from A2 (Figure 8a) to A1 (Figure 8c,d), passing through A2L (Figure 8b).

The analytical results, including further predictions of blends in the SI (see Figure S4), reveal the influence of selected compounds’ properties on the resulting mixtures.^109^ CO_2_, for instance, lowers both the GWP and flammability but increases the pressure and decreases the efficiency of the cycle. On the contrary, HCs, all with an A3 ASHRAE rating, lower GWP but increase flammability, while HFCs and HFOs exhibit a mixed set of attributes, revealing the necessity for novel combinations. From the results illustrated in Figure 8, it is observed that the choice of sHC/HO compounds is not a primary factor in determining flammability, as all of the considered carbon-based compounds are uniformly categorized under A3. Specifically, R1270 yields the lowest flammability, followed closely by R290 and R600a trailing behind. However, the choice and inherent influence of F-based compounds, ranging from A1 to A3, proved to have a more substantial impact on the resulting NFI of the assessed mixtures, with specific requirements on CO_2_ composition to achieve desired flammability indices, along with constraints on GWP. Certainly, the mapping of the flammability region, indicated in blue colormap, identified a sequence of refrigerants by ignition propensity, ordered as R125 (A1) < R134a (A1) < R1234yf (A2L) ∼ R1234ze(E) (A2L) < R1132a (A2) < R161 (A3).

In the case of A3 F-based refrigerants, a minimum mole composition of 90% CO_2_ is required within the blend to achieve an A1 flammability index, irrespective of the hydrocarbon portion. This requirement for CO_2_ could be reduced to 80% when A2-classified refrigerants (e.g., R1132a) are mixed with the same base constituents, leading to a refrigerant mixture with enhanced safety and maintenance features. However, utilizing A2L refrigerants like R1234yf or R1234ze(E)^110^ grants even greater flexibility in designing A1 blends, allowing the CO_2_ compositions to reach a minimum of 75% mol. Concurrently, the percentage of hydrocarbon is restricted to a maximum of 15 mol %, a constraint that could be elevated to 20–25 mol % when using A1-classified F-based refrigerants (e.g., R134a or R125). Indeed, within the formulation of refrigerant blends, it is consistently observed that the limiting factor governing the flammability of refrigerant blends invariably depends on the concentration of the carbon-based component. This constituent emerges as the maximum permissible concentration that can be added to the mixture in accordance with the NFI contours in Figure 8, a threshold beyond which flammability becomes a paramount concern. Even though A3 refrigerants bear a degree of limitation akin to that of R1270, R600a, or R290, their effect is typically more lenient, underscoring the critical role of hydrocarbons in the overall blend design. Overall, the predicted results, although exploratory in nature, align with reasonable expectations, thus reinforcing the reliability of the model, while also providing a structured framework to guide the formulation of refrigerant blends that reconcile flammability, environmental, and safety requirements.

## Conclusions

In this work, an ANN model has been developed to accurately predict the Normalized Flammability Index, as a measure of flammability, for a wide variety of refrigerants, including pure systems and blends involving compounds like CO_2_, HFCs, HFOs, sHCs, and HOs, among others. An extensive database including 20 pure-components, 1500 binary, and 1607 ternary blends has been compiled, with a wide spectrum of flammability characteristics, and used for ANN model training, testing, and validation. The developed ANN model employed 61 molecular descriptors based on the σ-profile obtained from COSMO-RS, with the optimal configuration of two hidden layers, with 14 and 24 neurons in each layer and tansig activation function after a strategic blend of systematic trial-and-error, iterative tuning, and cutting-edge optimization techniques. The resulting structure of the model has exhibited remarkable predictive power, with metrics such as an *R*^2^ of 0.999, RMSE of 0.1735, AARD% of 0.8091, SD_av_ of ±0.0434, and 81.3% of the data set with SDR of ±1. Moreover, there is a remarkable capacity to predict the behavior of additional 55 quaternary mixtures not included in model development, confirming the adaptability and general applicability of the model. Further validation has been achieved through the applicability domain analysis, demonstrating that 97.67% of the total 3D space was encompassed within the AD, a coverage that extends to 99.36% when considering borderline outliers. Additionally, the relative contributions of the 61 descriptors used as input parameters have been analyzed through the PaD method, identifying 23 significant descriptors collectively accounting for 90.98% of the total contribution. However, the reduction of the input descriptors to 23 has provided an average residual of 0.340, a 7-fold lower predictive precision compared to the more holistic approach employing all 61 descriptors. The developed methodology can therefore be applied to precisely forecast the flammability characteristics of novel untested mixtures, in line with industry needs, facilitating the screening of a diverse array of potential compounds toward the development and implementation of environmentally sustainable and safety-compliant refrigeration technologies. For this challenging task, a user-friendly layout within the Excel interface has been designed and is available in the Supporting Information, thereby aiding in the identification of refrigerants permissible in EU equipment in compliance with the most recent safety directives.

Capturing the essence of this part of our investigation, the main outcomes may be synthesized as follows: (1) NFI estimates align with expectations and thus settle a precedent for subsequent research of new, mildly to nonflammable fourth generation mixtures; (2) the formulation of such refrigerant blends confirmed the limiting factors leading to the identification of A1 regions within the search space, as per the NFI contour plots; (3) the hydrocarbon limit, set at 15 mol % for A2L + CO_2_ sequences in designing A1 blends, is projected to be increased to 20–25 mol % with the inclusion of A1 F-based refrigerants like R134a or R125; and (4) this study underscores the pivotal role of CO_2_ in tempering the flammability of high-efficient yet combustible refrigerants such as R161, R290, R600a, or R1270, thereby unlocking the path to high-energy-efficient systems with minimized safety risks. In summary, this research represents a milestone in the utilization of machine learning in the ever-evolving field of flammability analysis, as it provides reliable predictions that can be applied in real-world scenarios, including the precise domains of safety analysis, risk assessment, and the optimization of industrial processes.

## Supporting Information Available

Figures and tables on the database array; σ-profiles of the refrigerants included in the same; RMSE and elapsed time of different ANN architectures; ternary contour plots of CO_2_-based blends (PDF)
Output layer function of the developed ANN (ZIP)

## List of Abbreviations

AARD: absolute average relative error
Act: actual data
AD: applicability domain
AD_coverage_: applicability domain coverage
ANN: artificial neural networks
ASHRAE: American Society of Heating, Refrigerating, and Air-Conditioning Engineers
b: bias
BRO: boundary response outliers
BSO: boundary structural outliers
COSMO-RS: conductor like screening model for realistic solvents
*d**: number of descriptors
def-TZVP: double-electron, triple-ζ valence with polarization
DFT: density functional theory
e: elementary charge
ET: elapsed time
EU: European Union
F: fluorinated
GWP: global warming potential
*h**: leverage threshold
HBA: hydrogen bond acceptor
HBD: hydrogen bond donor
HCs: hydrocarbons
HFCs: hydrofluorocarbons
HFOs: hydrofluoroolefins
*h*_*i*_: leverage values
HLX: hidden layer X
HOs: hydroolefins or alkenes
I: input
LM: Levenberg–Marquardt
Logsig: logistic sigmoid
ML: machine learning
MSE: mean squared error
*N*: number of observations
*N*_c_: number of components
NFI: normalized flammability index
O: output
ODP: ozone depletion potential
p: total number of samples in the training set
*p*_AD_: number of observations within the applicability domain
*P*(σ): sigma profile
*P*_aD_: partial derivatives method
Pred: predicted data
Purelin: linear
*R*^2^: coefficient of determination
radbas: radial basis
RC: relative contribution
ReLU: rectified linear unit
RMSE: root mean square error
s: seconds
*S*_σ_^P^: surface charge density for molecule *i*
*S*_σ_^M^: surface charge density for mixture *M*
SCF: self-consistent field
SD_av_: average standard deviation
SDR: standardized residuals
sHCs: saturated hydrocarbons or alkanes
SMILES: simplified molecular input line entry system
*T*_ad_: adiabatic flame temperature
Tansig: hyperbolic tangent
*w*: weight
*x*_*i*_: mole fraction of compound *i*
*z*_*i*_: descriptor row vector for molecule *i*
*Z*: descriptor matrix associated with the training set

## Formulas, Units, and Greek Symbols

Å: Ångström
degree of fluorination
μ: learning rate parameter
π̅: normalized flammability index
# HL: number of hidden layers
# *N*_HL_: number of neurons in each hidden layer
# *S*_σ_: number of sigma profiles as inputs
σ^2^: residual variance
σ: specific charge density
