# Supplementary material for: Mapping the Flammability Space of Sustainable Refrigerant Mixtures through an Artificial Neural Network Based on Molecular Descriptors
Source: ACS Sustain Chem Eng. 2024 Jul 23;12(31):11561–77. doi: 10.1021/acssuschemeng.4c01961 (PMC11304399; doi:10.1021/acssuschemeng.4c01961)
Supplement: Supplementary file 1 — sc4c01961_si_001.pdf [file sc4c01961_si_001.pdf]

Supporting Information

Mapping the Flammability Space of Sustainable Refrigerant Mixtures Through an Artificial Neural Network based on Molecular Descriptors

Carlos G. Albà[1], Ismail I.I. Alkhatib[2], Lourdes F. Vega[2*], Fèlix Llovell[1*]

[1]Department of Chemical Engineering, ETSEQ, Universitat Rovira i Virgili (URV), Campus Sescelades, Av. Països Catalans 26, 43007, Tarragona, Spain.
[2]Research and Innovation Center on CO_2 and Hydrogen (RICH Center) and Department of Chemical and Petroleum Engineering, Khalifa University, PO Box 127788, Abu Dhabi, United Arab Emirates.

[*] Corresponding authors. *E-mail address*: lourdes.vega@ku.ac.ae; felix.llovell@urv.cat

Number of figures: 4 (Figures S1-S4)

Number of tables: 1 (Table S1)

Number of pages: 9 (S1-S9)

General Binaries				
HFC+HFC	HFO+HFO	sHC+sHC	HO+HO	RE170+R744
HFC+HFO	HFO+sHC	sHC+HO	HO+R744	
HFC+sHC	HFO+HO	sHC+R744	HO+RE170	
HFC+HO	HFO+R744	sHC+RE170		
HFC+R744	HFO+RE170			
HFC+RE170				Total

General Binaries				
402	144	30	0	0
611	29	3	0	
74	0	0	1	
1	91	2		
111	0			
1				1500

General Ternaries									
HFC+HFC+HFC	HFO+HFO+HFO								Total
HFC+HFC+HFO	HFC+HFC+sHC	HFC+HFC+HO	HFC+HFO+HFO	HFC+HFO+sHC	HFC+HFO+HO	HFO+HFO+sHC			
HFC+sHC+sHC	HFC+sHC+HO	HFO+HFO+HO	HFO+sHC+sHC	HFO+sHC+HO	sHC+sHC+HO				
R744+HFC+HFC	R744+HFC+HFO	R744+HFC+sHC	R744+HFC+HO	R744+HFO+HFO	R744+HFO+sHC	R744+HFO+HO	R744+sHC+sHC	R744+sHC+HO	R744+HO+HO
RE170+HFC+sHC									

General Ternaries									
127	24								1607
374	110	53	221	127	72	21			
18	35	7	9	13	1				
88	146	52	22	36	29	14	0	7	0
1									

Figure S1. *Database array for binary and ternary blends used in the developed ANN model.*

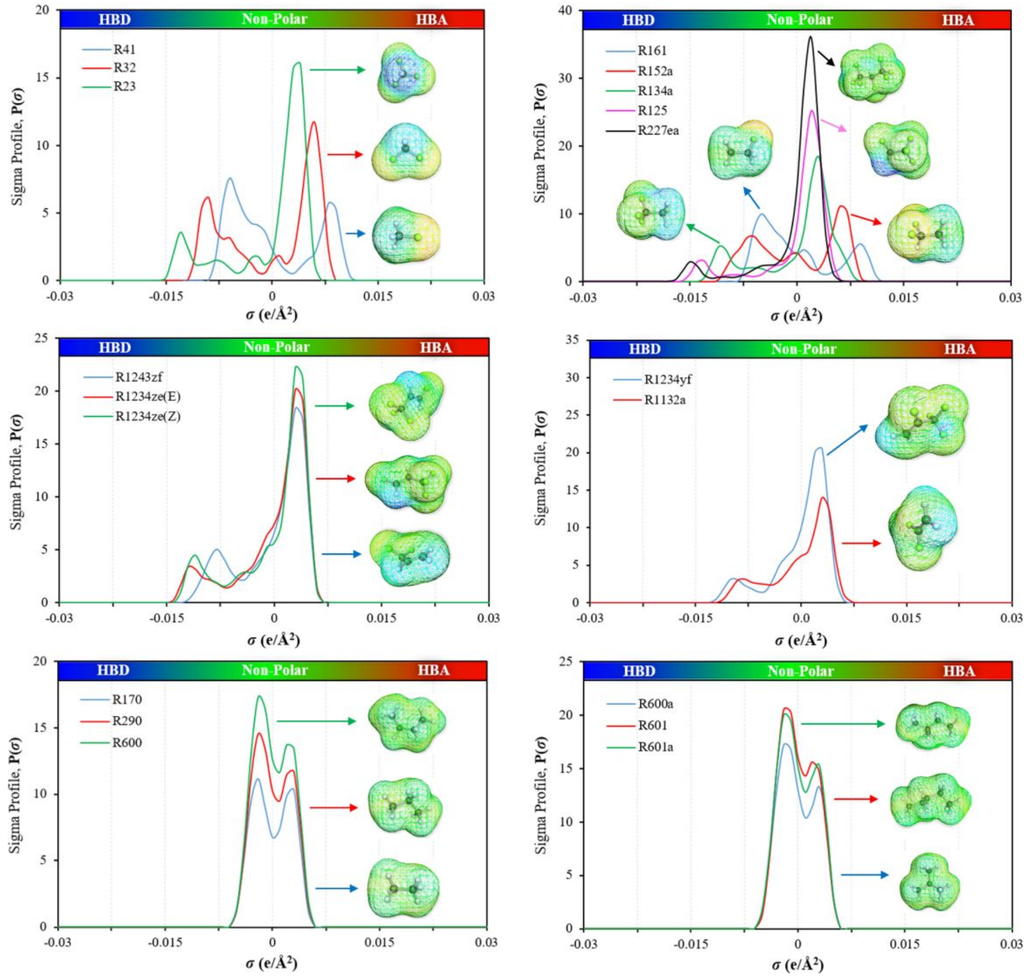

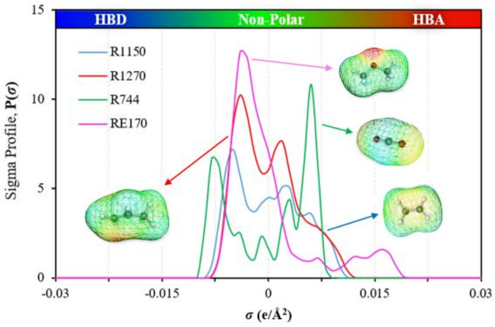

Figure S2. *σ-profiles of the 23 single-component refrigerants used in this work.*

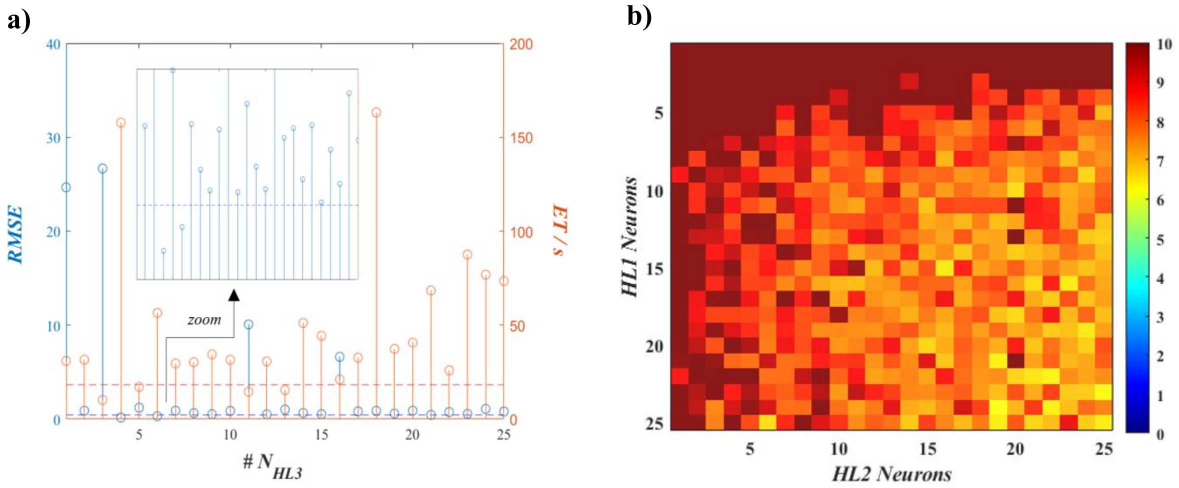

Figure S3. ***a)*** *RMSE and elapsed time per epoch for [61 (I) × 14 (HL1) × 24 (HL2) × X (HL3) × 1 (O)]) ANN architecture as a function of number of neurons in third hidden layer (HL3), and* ***b)*** *Mapping of the RMSE variation in relation to the number of neurons in hidden layers 1 and 2 when using 8 input molecular descriptors.*

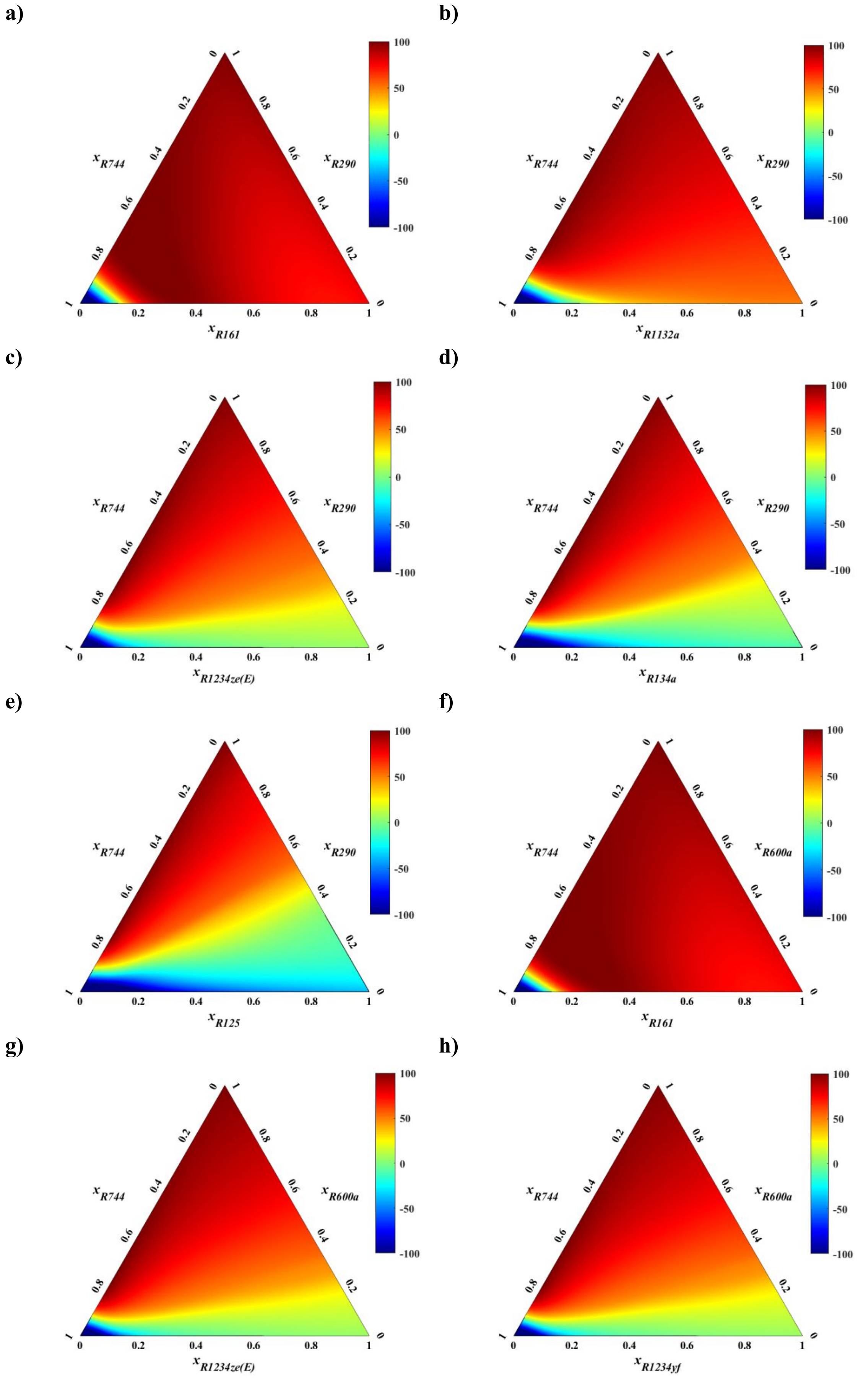
a)
x_{R744}
x_{R290}
x_{R161}
b)
x_{R744}
x_{R290}
x_{R1132a}
c)
x_{R744}
x_{R290}
$x_{R1234ze(E)}$
d)
x_{R744}
x_{R290}
x_{R134a}
e)
x_{R744}
x_{R290}
x_{R125}
f)
x_{R744}
x_{R600a}
x_{R161}
g)
x_{R744}
x_{R600a}
$x_{R1234ze(E)}$
h)
x_{R744}
x_{R600a}
$x_{R1234yf}$
100
50
0
-50
-100
0
0.2
0.4
0.6
0.8
1

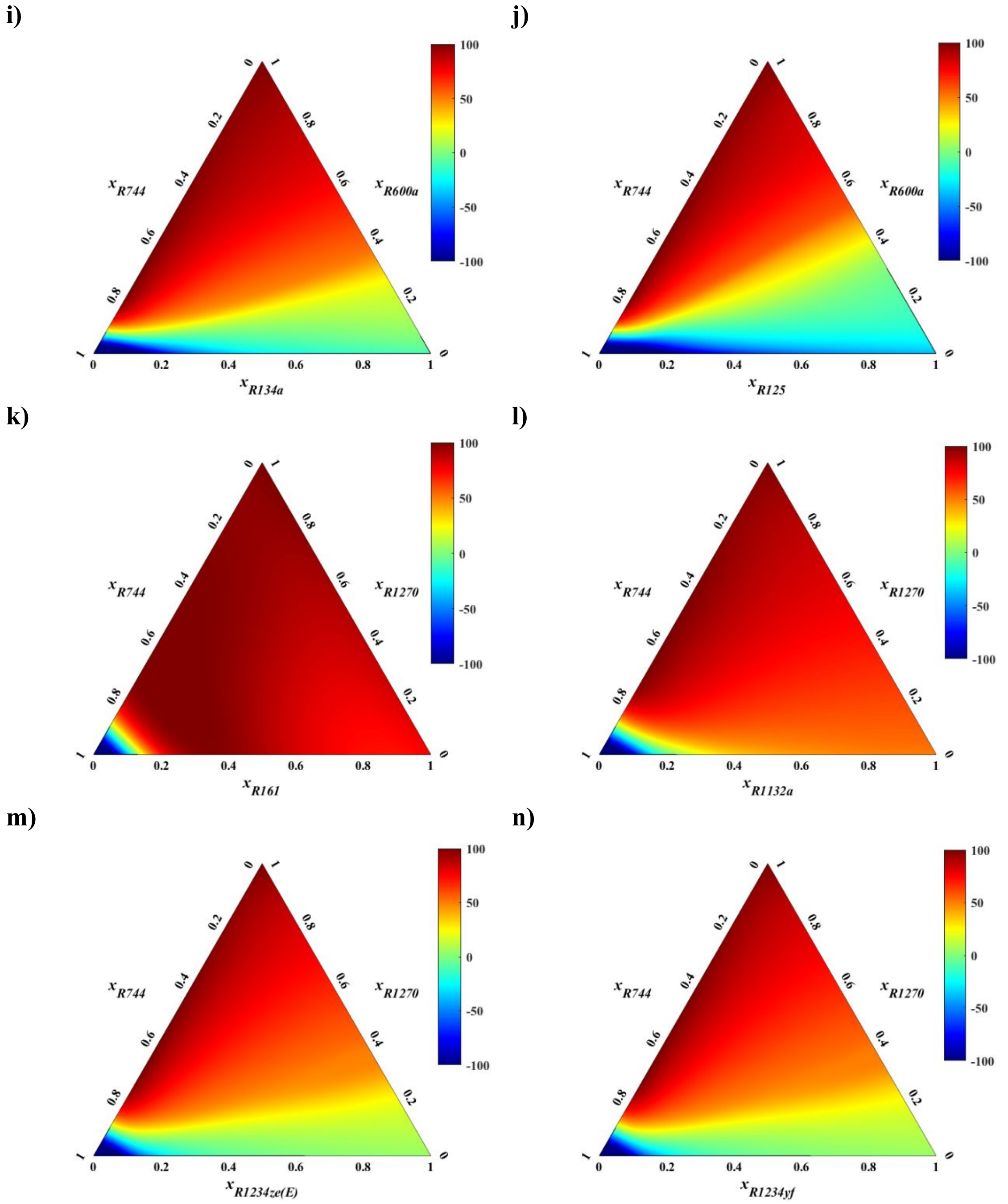

Figure S4. *Ternary contour plots generated by multitask ANN, depicting predictions of NFI for various CO_2 blends, including* ***a)*** *R290 + R161,* ***b)*** *R290 + R1132a,* ***c)*** *R290 + R1234ze(E),* ***d)*** *R290 + R134a,* ***e)*** *R290 + R125,* ***f)*** *R600a + R161,* ***g)*** *R600a + R1234ze(E),* ***h)*** *R600a + R1234yf,* ***i)*** *R600a + R134a,* ***j)*** *R600a + R125,* ***k)*** *R1270 + R161,* ***l)*** *R1270 + R1132a,* ***m)*** *R1270 + R1234ze(E), and* ***n)*** *R1270 + R1234yf.*

Table S1. *Developed ANN's weights and biases in addition to the output function enabling external predictions.*

	Hidden Layer 1													
	H_1	H_2	H_3	H_4	H_5	H_6	H_7	H_8	H_9	H_{10}	H_{11}	H_{12}	H_{13}	H_{14}
S_1														
S_2	0	0	0	0	0	0	0	0	0	0	0	0	0	0
S_3	0	0	0	0	0	0	0	0	0	0	0	0	0	0
S_4	0	0	0	0	0	0	0	0	0	0	0	0	0	0
S_5	0	0	0	0	0	0	0	0	0	0	0	0	0	0
S_6	0	0	0	0	0	0	0	0	0	0	0	0	0	0
S_7	0	0	0	0	0	0	0	0	0	0	0	0	0	0
S_8	0	0	0	0	0	0	0	0	0	0	0	0	0	0
S_9	0	0	0	0	0	0	0	0	0	0	0	0	0	0
S_{10}	0	0	0	0	0	0	0	0	0	0	0	0	0	0
S_{11}	0	0	0	0	0	0	0	0	0	0	0	0	0	0
S_{12}	0	0	0	0	0	0	0	0	0	0	0	0	0	0
S_{13}	0	0	0	0	0	0	0	0	0	0	0	0	0	0
S_{14}	0.8458	-2.8818	-1.0014	1.0280	0.0565	-2.4029	-1.8018	2.8284	-1.3958	-2.6598	-1.2253	1.5103	-2.4375	-0.4681
S_{15}	-0.2075	0.5565	-0.4336	-0.3225	0.8986	-1.4568	0.0141	-0.5083	-0.6832	-0.9620	0.0730	-0.5960	-0.7045	-0.3797
S_{16}	-0.0420	-0.9716	0.1980	0.2326	0.4817	-0.5778	0.1160	-0.1190	-0.2024	-0.8362	-0.4973	0.3534	-0.0101	-0.2451
S_{17}	-0.1242	-0.6831	-0.3918	0.0612	0.4498	0.9127	0.3810	0.1004	-0.1382	0.0039	-0.2620	0.4858	0.0129	0.3271
S_{18}	-0.2648	0.7208	0.0489	-0.4739	0.3182	-0.9774	-0.4748	0.6585	-0.0579	0.1070	0.3020	0.0031	-0.2046	-0.0182
S_{19}	0.5436	-0.2822	0.1789	0.0545	-0.3789	0.3121	0.2268	-0.1822	-0.0223	-1.0864	0.2265	-0.1448	-0.6931	-0.1322
S_{20}	-0.0544	-0.1080	-0.2226	0.1345	-0.2125	0.8676	0.1626	-0.4861	0.3767	0.2059	-0.4288	0.0671	0.2071	0.0781
S_{21}	-0.1340	0.1226	-0.0720	-0.1219	0.1343	-0.6544	-0.3892	-0.1006	0.3139	0.4365	-0.2181	-0.0986	0.5160	0.0535
S_{22}	0.3013	-0.0026	0.3618	0.1885	-0.4095	0.7669	0.2337	0.0294	-0.8519	-0.8500	0.9069	0.0183	-0.5814	0.0328
S_{23}	0.0989	-0.7800	0.3459	0.1297	-0.4147	0.8101	-0.1587	-0.3154	-0.0937	0.6298	-1.1329	0.5140	0.0257	0.2170
S_{24}	0.0783	0.3142	-0.4355	-0.0218	-0.0870	-0.6036	-0.0510	-0.1005	0.7059	0.6007	0.2707	-0.4815	0.0817	-0.2435
S_{25}	-0.3056	1.3469	-0.0593	-0.0752	0.0691	-0.4876	-0.3930	0.2413	-0.6005	0.5594	1.0563	-0.3262	0.2841	0.0201
S_{26}	-0.6668	-0.5203	0.1390	0.1239	0.0474	0.4045	0.0559	0.1043	-0.6262	1.6126	-0.4221	0.4299	0.3607	-0.0431
S_{27}	0.3305	-0.0070	-0.1061	0.2185	0.0385	0.3718	0.0391	-0.0966	0.1564	-1.2582	0.5244	-0.0936	-0.0046	0.1240
S_{28}	-0.1911	0.2889	-0.1037	0.0308	-0.3479	-0.7942	-0.3377	0.2884	-0.0728	0.5285	-0.5527	-0.1052	0.3868	-0.0964
S_{29}	-0.7246	-0.3970	-0.3013	0.1151	-0.5175	-0.3552	0.4574	0.1324	-0.5002	2.5340	0.2442	0.0971	0.3455	-0.5602
S_{30}	-0.0133	0.4186	0.5083	0.2190	0.3684	1.0784	-0.5578	-0.2062	-0.6011	-1.1690	0.4780	0.2896	0.0142	0.8000
S_{31}	0.3900	-0.2749	0.0112	-0.4003	0.2787	-0.1079	0.0516	0.0322	0.8641	-0.8075	-0.8632	-0.1145	0.0146	-0.0451
S_{32}	0.3012	0.1685	-0.3948	0.0012	-0.2608	-0.3707	0.3654	-0.5010	0.3648	0.0772	0.2757	-0.2886	-0.0134	-0.5134

**S_{33}**	-0.3695	0.2555	0.3861	0.3996	0.1019	0.7585	-0.3733	-0.1764	-0.4813	-0.0184	0.2333	0.1530	0.2254	0.6138
**S_{34}**	0.1128	-0.5109	-0.0800	-0.5002	0.3694	-0.6640	0.3490	0.5210	0.2498	-0.2953	-0.1726	0.0795	-0.3850	-0.2771
**S_{35}**	0.1812	0.7339	-0.1567	0.1618	-0.2162	-0.2073	-0.1199	-0.3285	-0.1126	-0.0944	0.4139	-0.1916	0.1889	-0.1669
**S_{36}**	-0.4721	-0.6985	0.2856	0.2656	0.1084	0.5206	-0.0305	-0.2051	0.1354	0.7381	-0.5957	0.1159	0.5055	0.3921
**S_{37}**	0.2246	0.4807	-0.3618	-0.5902	0.2549	-0.6401	0.1568	0.5597	-0.1226	-0.9058	0.8417	0.0656	-0.3964	-0.0706
**S_{38}**	0.0929	0.0432	0.1408	0.2417	-0.0592	-0.1697	-0.0024	-0.3407	-0.0637	0.2076	-0.4002	-0.0228	-0.0229	-0.2159
**S_{39}**	0.2257	-1.1726	0.4945	0.2524	-0.0465	0.8416	0.2879	-0.5317	0.9494	0.2598	-0.8329	-0.0505	0.0145	0.2459
**S_{40}**	0.6905	0.2610	-0.1590	-0.2942	-0.0081	0.2651	0.2368	0.0789	0.1985	-1.6810	0.8810	0.0659	-0.5303	0.1440
**S_{41}**	0.4459	-0.3950	0.3738	-0.1648	0.3883	-0.9344	-0.1096	-0.0361	-0.9516	-0.6250	-1.0354	0.7487	-0.4512	0.0779
**S_{42}**	0.2501	0.1639	0.6585	-0.4493	-0.0411	-1.0826	-0.8020	0.3601	-0.7561	-0.5254	-1.1717	-0.1687	-0.3834	-0.3302
**S_{43}**	-0.7899	0.6348	0.1914	0.6987	-0.5303	-0.4871	0.3696	0.0225	0.3617	-0.2846	0.1156	0.3481	-0.3268	-0.6428
**S_{44}**	0.6794	-0.7828	-0.0959	-0.1586	0.7576	-0.4573	0.1760	-0.6900	-0.0469	-0.7902	-0.5326	-0.7873	0.4142	-0.0299
**S_{45}**	0.2992	0.4066	0.2741	-0.6989	-0.6230	-0.1659	0.5104	0.1735	-0.0891	0.4978	0.0956	0.2860	-0.3166	0.3128
**S_{46}**	-0.4264	-0.0579	0.2603	0.4627	0.6417	0.1776	-0.2646	0.4489	0.5059	0.2361	0.3559	0.5683	-0.1049	-0.2904
**S_{47}**	0.0468	-0.0539	-0.7060	0.2142	-0.0446	-0.5711	0.3175	-0.1118	-0.4560	0.0912	-0.2672	-0.4591	-0.1369	-0.3181
**S_{48}**	-0.6722	-0.2767	0.5550	-0.0732	0.6714	-0.7642	-0.0076	0.5971	0.1949	-0.1274	0.2126	0.4536	-0.3957	-0.2523
**S_{49}**	-0.9666	0.2498	-0.0633	0.7506	0.1629	0.3114	1.2805	0.0397	-0.3651	-0.0011	0.2802	0.5257	0.5121	0.7068
**S_{50}**	0.2117	4.8667	-6.7478	5.8320	-4.7707	-0.8295	6.7468	5.7124	8.0412	0.2563	5.8112	5.9861	1.3188	-7.6719
**S_{51}**	0	0	0	0	0	0	0	0	0	0	0	0	0	0
**S_{52}**	0	0	0	0	0	0	0	0	0	0	0	0	0	0
**S_{53}**	0	0	0	0	0	0	0	0	0	0	0	0	0	0
**S_{54}**	0	0	0	0	0	0	0	0	0	0	0	0	0	0
**S_{55}**	0	0	0	0	0	0	0	0	0	0	0	0	0	0
**S_{56}**	0	0	0	0	0	0	0	0	0	0	0	0	0	0
**S_{57}**	0	0	0	0	0	0	0	0	0	0	0	0	0	0
**S_{58}**	0	0	0	0	0	0	0	0	0	0	0	0	0	0
**S_{59}**	0	0	0	0	0	0	0	0	0	0	0	0	0	0
**S_{60}**	0	0	0	0	0	0	0	0	0	0	0	0	0	0
**S_{61}**	0	0	0	0	0	0	0	0	0	0	0	0	0	0
**b_1**	0.1353	-2.2195	-0.1200	-3.2270	1.4274	1.6435	0.5930	-0.5451	2.4942	-2.4254	-3.1677	-0.7024	-0.9291	-0.0244

Hidden Layer 2												
	**HH$_1$**	**HH$_2$**	**HH$_3$**	**HH$_4$**	**HH$_5$**	**HH$_6$**	**HH$_7$**	**HH$_8$**	**HH$_9$**	**HH$_{10}$**	**HH$_{11}$**	**HH$_{12}$**
**H$_1$**	-0.9736	-0.9666	-0.2496	-1.0789	-1.7361	0.8504	0.1465	-0.0989	0.4060	0.6723	-0.0490	0.5446
**H$_2$**	-0.1403	-0.1408	-0.5551	-0.3780	0.5694	-0.1034	-0.2616	0.5531	1.2341	-0.1411	0.1482	1.0281
**H$_3$**	0.9141	-0.5179	0.5083	0.3097	1.1655	-0.1528	-1.0784	0.0616	-0.8161	-0.7366	1.1396	-0.4722
**H$_4$**	-0.0151	0.8341	0.1691	-0.1395	-0.6732	-1.1298	-0.0353	-0.4870	-0.0799	-0.7731	0.0268	-0.0450
**H$_5$**	-0.4041	-0.3617	-1.2703	-0.7659	-1.3013	-0.1552	0.0511	0.0172	1.1414	0.6586	-0.7818	1.6466
**H$_6$**	-1.5985	0.8163	0.0733	-0.9836	0.1540	-0.0187	0.3907	-1.3976	-0.8624	-0.8776	-0.6272	-0.4068
**H$_7$**	-0.3881	0.6095	0.0643	-0.3551	0.6241	0.4422	0.0965	0.0174	-0.4386	-0.3572	0.2783	0.0528
**H$_8$**	-0.4478	-1.3546	0.3756	-0.3951	-0.5737	-0.4195	0.1443	-0.2789	-0.5951	-0.3784	-0.9224	-0.9357
**H$_9$**	0.0216	-0.3010	-0.1021	-1.6722	-1.3522	0.2581	-0.5431	0.2483	0.4768	-0.3070	0.0820	0.5090
**H$_{10}$**	1.0890	-0.2895	0.4189	0.7706	-0.8508	0.1307	-0.1802	0.3936	0.2069	0.3856	0.5033	-0.0787
**H$_{11}$**	-0.3058	0.0690	0.2709	0.5627	-1.0444	-0.7898	-0.6406	-1.1523	0.0305	0.4805	0.3936	-1.7033
**H$_{12}$**	-0.0054	0.0633	-0.6880	1.9224	-0.1050	-1.0584	0.1281	-0.4816	0.6330	1.2022	-0.1686	1.1883
**H$_{13}$**	1.1005	-0.8841	-0.0160	0.5523	1.0234	0.5340	-0.7862	0.0010	0.9427	-0.1897	-0.8109	2.0533
**H$_{14}$**	0.4210	-0.7273	-0.5702	0.5410	0.9315	-0.6775	-0.0949	1.2565	-0.9530	-0.2399	-0.5506	-0.6708
**b$_2$**	1.4354	1.2921	1.3947	1.1705	1.4242	-0.1033	1.2042	-0.1800	1.3800	0.2428	0.6424	0.2635

Hidden Layer 2 (continuation)												
	**HH$_{13}$**	**HH$_{14}$**	**HH$_{15}$**	**HH$_{16}$**	**HH$_{17}$**	**HH$_{18}$**	**HH$_{19}$**	**HH$_{20}$**	**HH$_{21}$**	**HH$_{22}$**	**HH$_{23}$**	**HH$_{24}$**
**H$_1$**	0.1277	0.3547	-0.5744	-0.1646	-0.6186	-0.2490	1.2647	-0.1917	-0.2853	-0.0538	0.5955	1.0445
**H$_2$**	0.9935	0.4954	-0.1891	0.3422	-1.5464	-1.4037	0.5618	0.4680	0.3774	-0.0625	0.6502	0.7284
**H$_3$**	-0.3273	0.4333	1.0209	-1.0051	-0.0063	0.5486	-1.0028	0.5176	1.5267	0.1244	1.0048	1.6874
**H$_4$**	-1.7088	-0.7940	0.1285	-0.0501	0.6612	-0.7574	0.0097	0.0305	0.5573	-0.1345	0.1096	-0.5886
**H$_5$**	-0.5840	0.1511	0.2030	-0.0389	-1.4057	0.2793	0.5602	0.3263	-0.3398	0.0903	-0.7871	1.0246
**H$_6$**	-0.4323	0.0185	-0.3368	0.3474	2.6329	1.9890	0.9805	0.3673	0.7953	0.9229	0.8087	-0.6728
**H$_7$**	0.1431	0.9938	0.4666	-0.2076	1.1721	0.1023	-0.6531	0.4515	0.2532	0.1245	0.1330	-0.8273
**H$_8$**	1.5987	-0.4215	-1.5950	0.0558	0.5198	0.1864	-1.6606	-0.3550	-0.4185	0.9727	0.0257	0.1225
**H$_9$**	-0.0561	0.8727	-0.5854	-0.7616	-0.1307	1.3431	1.0849	0.3158	0.3652	0.1168	-1.2679	-0.5087
**H$_{10}$**	-0.1359	-0.4421	1.9128	-0.2665	2.8886	0.9070	-0.0831	-0.6031	-0.2184	-1.5533	-0.3674	-0.1274
**H$_{11}$**	1.8617	-1.5216	-0.6076	-0.4293	-0.4676	-0.0239	0.6304	0.3375	-0.4912	-0.0163	0.1674	-0.9631
**H$_{12}$**	0.0334	0.0001	-0.4204	0.2932	0.7984	1.0063	0.4212	1.0249	0.7497	0.7885	0.2674	-0.8087
**H$_{13}$**	-0.1975	-1.1089	-0.0487	1.1674	-0.5084	0.0914	-1.5168	-0.0703	0.1331	0.6427	-0.7398	0.2498
**H$_{14}$**	-0.2919	1.3184	-1.1254	1.6070	1.0834	-0.9243	-1.2359	-0.0689	-0.2937	0.7242	0.6219	0.0282
**b$_2$**	0.4595	1.6282	-0.0898	-0.8157	0.0004	-1.7517	0.6497	-0.5375	-1.0831	-2.0170	1.4198	2.5383

Output Layer
$NFI_{predicted} = 0.248097(HH_1) - 0.319464(HH_2) + 0.162838(HH_3) + 0.008849(HH_4) + 1.808459(HH_5) + 0.085165(HH_6) + 0.205977(HH_7)$ $+ 0.264793(HH_8) - 0.620327(HH_9) - 0.029470(HH_{10}) - 0.221097(HH_{11}) + 0.488371(HH_{12}) - 0.178911(HH_{13}) - 0.272909(HH_{14})$ $+ 0.123859(HH_{15}) - 0.151293(HH_{16}) + 0.029668(HH_{17}) + 0.055991(HH_{18}) - 0.209633(HH_{19}) + 0.237560(HH_{20}) + 0.126407(HH_{21}) + 0.166035(HH_{22}) + 0.1$[illegible] $- 0.433878$
